# Computational framework for single-cell spatiotemporal dynamics of optogenetic membrane recruitment

**DOI:** 10.1016/j.crmeth.2022.100245

**Published:** 2022-07-06

**Authors:** Ivan A. Kuznetsov, Erin E. Berlew, Spencer T. Glantz, Pimkhuan Hannanta-Anan, Brian Y. Chow

**Affiliations:** 1 Department of Bioengineering, University of Pennsylvania, Philadelphia, PA 19104, USA; 2 Department of Biomedical Engineering, School of Engineering, King Mongkut's Institute of Technology Ladkrabang, Bangkok 10520, Thailand

**Keywords:** single cell, finite element model, finite element analysis, optogenetics, peripheral membrane protein

## Abstract

We describe a modular computational framework for analyzing cell-wide spatiotemporal signaling dynamics in single-cell microscopy experiments that accounts for the experiment-specific geometric and diffractive complexities that arise from heterogeneous cell morphologies and optical instrumentation. Inputs are unique cell geometries and protein concentrations derived from confocal stacks and spatiotemporally varying environmental stimuli. After simulating the system with a model of choice, the output is convolved with the microscope point-spread function for direct comparison with the observable image. We experimentally validate this approach in single cells with BcLOV4, an optogenetic membrane recruitment system for versatile control over cell signaling, using a three-dimensional non-linear finite element model with all parameters experimentally derived. The simulations recapitulate observed subcellular and cell-to-cell variability in BcLOV4 signaling, allowing for inter-experimental differences of cellular and instrumentation origins to be elucidated and resolved for improved interpretive robustness. This single-cell approach will enhance optogenetics and spatiotemporally resolved signaling studies.

## Highlights

• Microscopy data suffer from variability due to cell-intrinsic/extrinsic factors
• We describe a framework for spatiotemporal analysis of single-cell microscopy
• We use the approach to model BcLOV4, an optogenetic membrane recruitment system
• The approach resolves inter-experimental differences and guides future tool design

## Motivation

Single-cell microscopy experiments, when paired with appropriate inducible control systems and physiological reporters, allow for precise probing of cell-wide spatiotemporal signaling dynamics. Interpretation of these data and inter-experiment comparisons can be difficult, though, due to inherent variability both from cell-intrinsic factors (e.g., protein expression and subcellular localization) and cell-extrinsic factors (e.g., instrumentation-dependent diffractive phenomena). To address these challenges, we created an *in silico* framework to study single-cell signaling via microscopy experiments. We apply this framework toward dynamic membrane recruitment of a cytosol-sequestered protein, a powerful approach to control signaling, specifically focusing on BcLOV4 optogenetic technology. This framework can improve single-cell signaling analysis and guide forward the design of optogenetic tools.

## Graphical abstract

**Figure:**
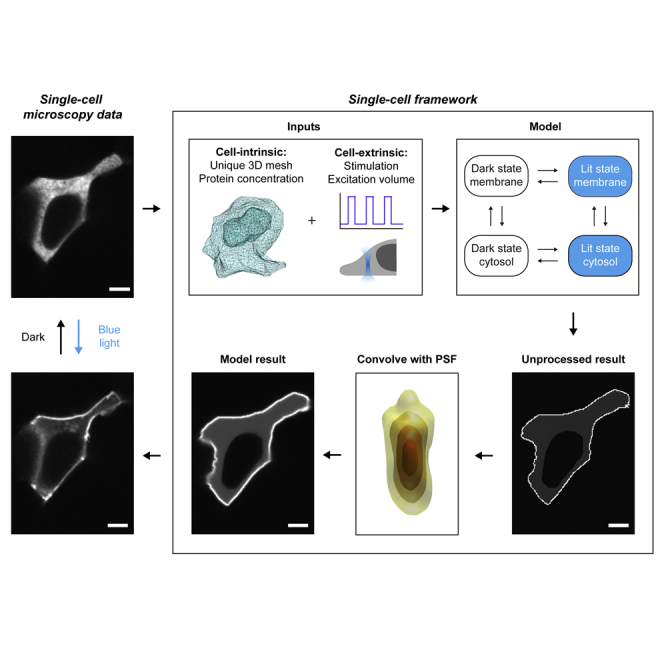

## Teaser

Kuznetsov et al. describe a computational framework for analyzing cell signaling in single-cell microscopy and use it to study BcLOV4 optogenetic membrane recruitment. By accounting for inter-experimental differences of cell and instrumentation origin, the approach allows for robustness to inter-experimental variability. This method will enhance spatiotemporally resolved signaling studies.

## Introduction

Quantitative understanding of subcellular signaling dynamics is key to cell physiology and predicting cell behaviors. To this end, a growing number of increasingly complex models and model design tools have been reported, e.g., Virtual Cell (VCell), MCell/CellBlender, and COMSOL packages, among others (Stiles et al., 1996; Schutter, 2000; Kerr et al., 2008; Moraru et al., 2008; Menshykau, 2013; Hallett et al., 2016). While single-cell analyses can reveal the physiological underpinnings of heterogeneous cell behaviors, cell models often do not accurately reflect such heterogeneity. They typically are fit to population-average phenotypes and apply simplifying assumptions regarding cell geometry and protein expression (Cheong et al., 2010; Handly et al., 2016). Thus, validated computational frameworks to predict spatiotemporally resolved subcellular signaling dynamics of unique single cells can advance quantitative biology. Accounting for single-cell experimental variability from extrinsic or non-cellular factors, such as instrumentation-dependent diffractive phenomena in an optogenetics experiment, would further improve comparisons between model and experiment.

**Figure 1:**
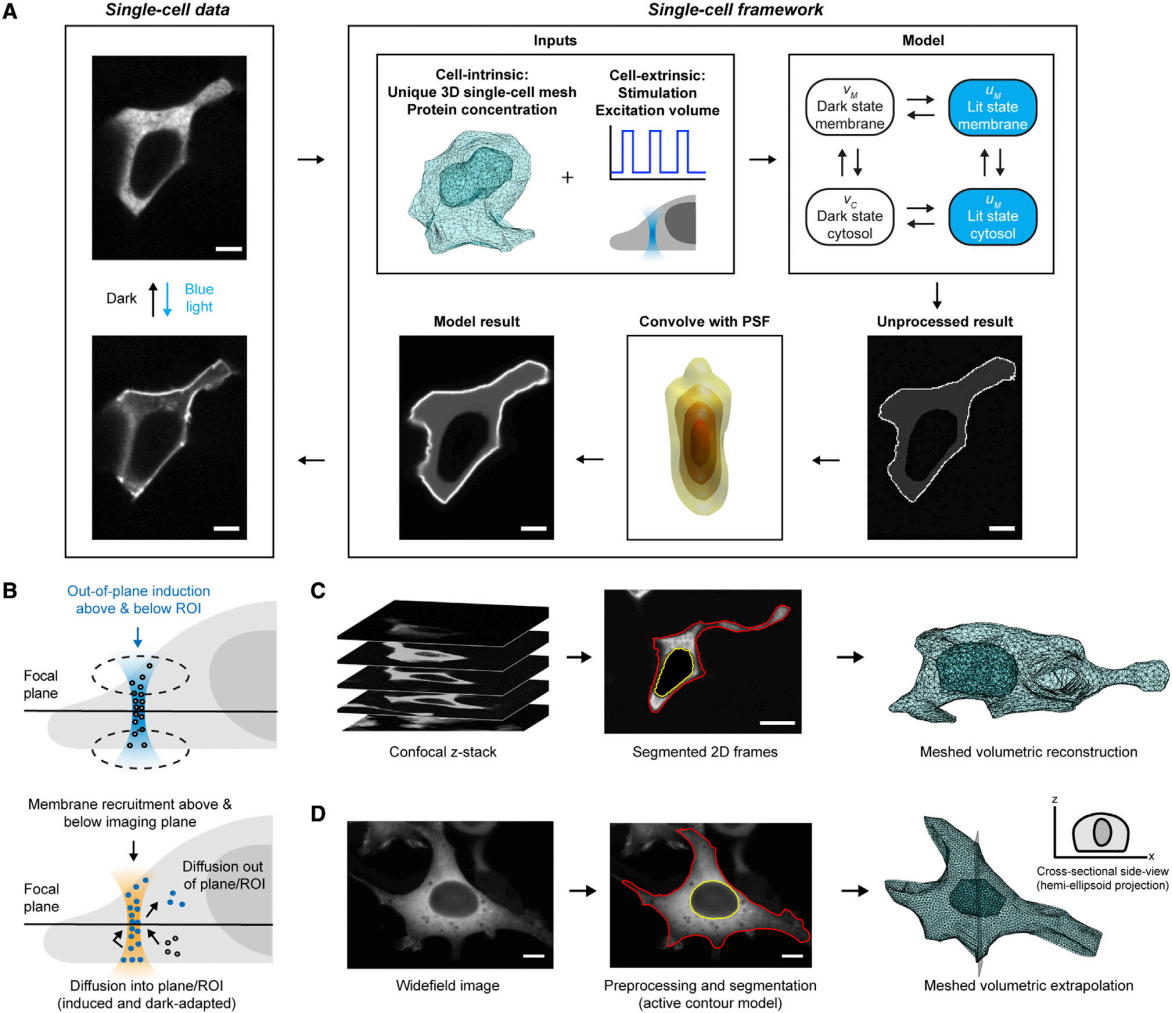
Single-cell data-unique simulation framework BcLOV4 refers to BcLOV4-mCherry. (A) Workflow. BcLOV4-expressing HEK cells were imaged with a confocal (shown) or widefield microscope. Framework data-unique inputs were in two categories: cell-intrinsic (unique to the single cells) and cell-extrinsic (attributable to experimental parameters and hardware). Cells were reconstructed in 3D, meshed using the initial membrane and nucleus contours, and initialized with the cytoplasmic protein concentration. Cell-extrinsic inputs were determined by the experimental stimulation paradigm (i.e., light intensity, spatial patterning, duration, and duty cycle), and microscope excitation volume. The 4-state bulk-surface model predicted the spatiotemporal behavior of that cell. The unprocessed results were convolved with the microscope point-spread function (PSF) so the model result could be directly compared with experimentally observed data. (B) Motivation for volumetric approach to capture geometric and diffractive effects unique to individual cells (e.g., surface area/volume ratio) and experiment conditions (e.g., hardware PSF). Diffraction-limited excitation of a region of interest (ROI) and post-induction imaging are schematized for a laser-scanning confocal microscope. Imaging effects include membrane recruitment above/below the excitation plane, diffusion of activated protein in/out of the imaging plane, and diffusion of dark-adapted protein into the ROI. (C) 3D mesh generation from interpolated confocal z stacks to reconstruct single-cell-unique morphology. (D) 3D mesh generation from widefield images. Hemi-ellipsoid projection was used to extrapolate a volume (i.e., flat bottom to account for cell∷dish contact). Scale bars, 5 μm (A) and 10 μm (B and C).

To these ends, we created a modular *in silico* framework for spatiotemporally resolved study of single-cell signaling in microscopy experiments. The framework is composed of (1) cell-intrinsic inputs that reflect unique cell geometry and protein concentration, (2) cell-extrinsic inputs to reflect experiment-/instrument-specific conditions, (3) a mathematical model to predict the subcellular spatiotemporal response, and (4) an output point-spread function (PSF) correction to allow direct comparison between a unique model and corresponding data (Figure 1). This framework accurately recapitulates non-equilibrium dynamics across a unique three-dimensional (3D) cell and mirrors optical hardware-dependent experimental conditions used to generate single-cell data. Thus, it will enhance our biophysical understanding of signaling processes and guide the onward design of spatiotemporally complex signaling outputs.

We apply this framework to study dynamic membrane recruitment of a cytosol-sequestered protein, a ubiquitous approach in optogenetics and related chemical techniques to control signaling (Inoue et al., 2005; Repina et al., 2017; Stanton et al., 2018; Hannanta-Anan et al., 2019). It can be mediated by an inducible heterodimerizing protein-protein interaction or by inducible protein-lipid interaction with the plasma membrane itself in a single-component system (He et al., 2017; Glantz et al., 2018; Li et al., 2021). Here, we study the single-component BcLOV4 that we have previously used for versatile blue light control over GTPase signaling (Hannanta-Anan et al., 2019; Berlew et al., 2020, 2021, 2022).

We employ a non-linear 3D finite element model (FEM). This is well suited to the complex geometries of mammalian cells and organelles, which are inherently highly irregular in morphology, size, protein expression, and protein distribution. It is unlike existing mathematical models of optogenetic membrane recruitment that have been idealized (symmetric two-dimensional [2D]), have been solved under steady-state conditions and/or have assumed linearity with respect to concentration, despite the fact that the 3D recruitment process is dynamic and increasingly non-linear with membrane binding site occupancy (Valon et al., 2015; Niu et al., 2016; de Beco et al., 2018; Natwick and Collins, 2021). In brief, model biophysical parameters were experimentally derived. For each cell, the mesh was autogenerated from cell images and then seeded with the initial protein concentration. Use of actual cell geometry and protein concentration ensured accurate prediction of the spatiotemporal signaling outputs in response to arbitrary light inputs. Furthermore, inclusion of instrument-dependent diffractive effects, by way of PSF correction of the simulated output, permitted direct correlation with its corresponding single-cell image data.

We use the framework to explore how cell-specific morphology, optogenetic protein-specific biophysical properties, and the hardware-specific optical properties of the stimulatory input all shape the resultant observed signaling output. These analyses successfully identified the origins of detection artifacts that confound data interpretation. To demonstrate generality, we also show how existing software tools can be integrated into the modular framework by implementing it in VCell to similar effect. This work establishes a foundation for further spatiotemporally accurate models of peripheral membrane protein signaling and shows how single-cell models can reflect and combat experimental heterogeneity for improved interpretive robustness.

## Results

BcLOV4 is a well-posed and simple test bed for validating the framework. Its optical stimulation can be modulated with subcellular precision, and the diffractive properties of the optical hardware are measurable when testing how well the framework accounts for cell-extrinsic and experiment-specific factors. As BcLOV4 directly binds the plasma membrane in a light-dependent manner, its minimal single-component nature simplifies modeling by limiting the set of equations and the terms/constants to be measured; it also streamlines experiments without the need to express and quantify multiple proteins in live cells. Henceforth, “BcLOV4” refers to the fluorescently labeled BcLOV4-mCherry, unless stated otherwise; also, “lit state” or “lit” refers to its photoactive state, and “dark state” or “dark” refers to dark-adapted protein.

There are several components of the framework implementation described herein: (1) the automated mesh generation from image volumes of corresponding cells; (2) the set of non-linear partial differential equations (PDEs) and associated custom solver; (3) the experimental measurement of biophysical constants in the model; and (4) the convolution of the microscope PSF to correlate the theoretical output to the experimental observable.

### Automated 3D mesh generation

The 3D mesh was necessary for biophysical accuracy. Since the stimulation volume has a non-negligible axial extent, the protein diffuses in three dimensions in and out of the imaging plane, to and from the membrane, and within the bulk cytosol (Figure 1B). Diffractive effects lead to inevitable contributions of cytosolic fluorescence to membrane fluorescence signal and vice versa (Figure 1B), which can confound data interpretation. This led us to generate a unique tetrahedral mesh for each experimental cell and construct a 3D FEM. Mesh generation was almost fully automated from fluorescence micrographs of HEK cells expressing BcLOV4. The shape of the plasma and nuclear membranes were reconstructed from confocal microscopy image stacks by interpolation in the z axis (Figure 1C), or were extrapolated by hemi-ellipsoid projections (i.e., adherent cells on flat-bottom substrates) when only one focal plane was available (Figure 1D). Mesh nodes were initialized with the corresponding protein concentration in the starting dark-adapted state.

### Bulk-surface reaction-diffusion system

**Figure 2:**
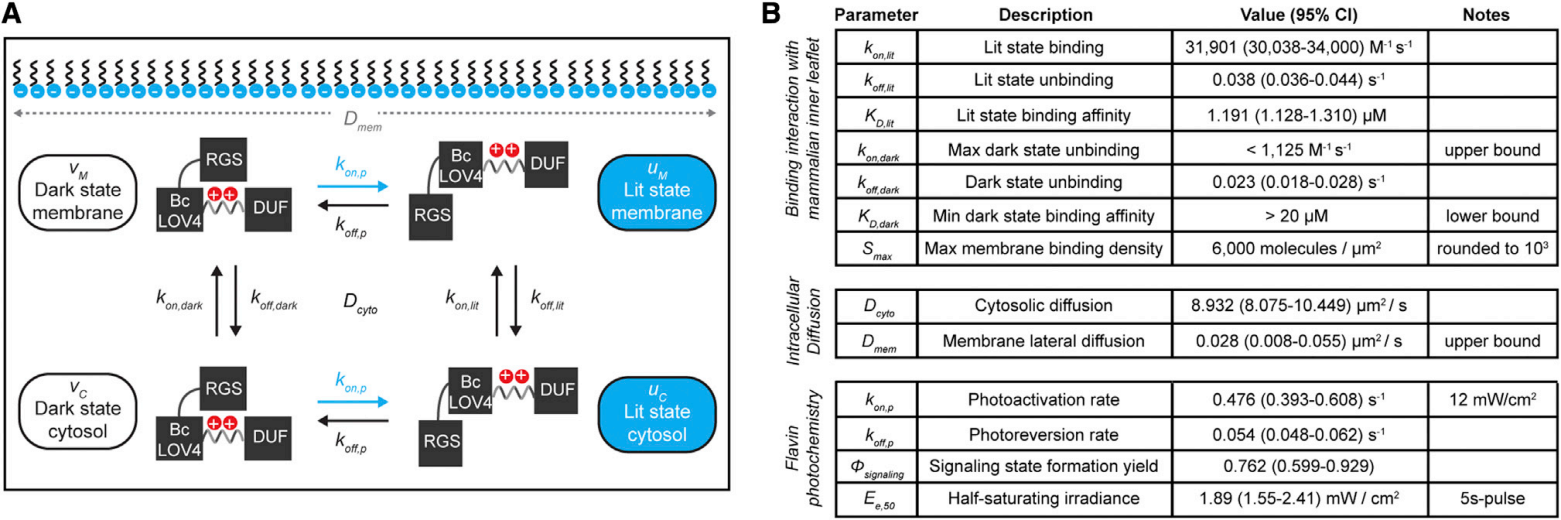
Experimentally determined biophysical constants BcLOV4 refers to BcLOV4-mCherry, unless stated otherwise. (A) Four-state bulk-surface reaction-diffusion model of BcLOV4 membrane recruitment. (B) Binding constants and rates directly measured in live HEK single cells by cytoplasmic depletion fluorescence microscopy. Diffusion rates were determined by FRAP microscopy (Figures S3 and S4). When needed for feasibility, upper/lower bounds were chosen as conservative values, as noted. Flavin photochemistry was measured by absorbance spectroscopy using FPLC-purified bacterial-expressed recombinant protein without fused mCherry (STAR Methods; λ = 455 nm). The parameter set, along with fluorescence-derived absolute protein concentration (Figure S1) and measured hardware PSF (STAR Methods), is sufficient to fit the global minimum of the proposed single-cell models.

We modeled the recruitment process as a four-state system: (1) photoactivated lit-state and cytosolic ($u_{C}$); (2) dark-adapted and cytosolic ($v_{C}$); (3) lit-state and membrane-localized ($u_{M}$); or (4) dark-adapted and membrane-localized ($v_{M}$) (Figure 2A). The $v_{M}$ state includes the period that BcLOV4 stays associated with the membrane (∼60 s) after thermal reversion of the flavin photoadduct (Glantz et al., 2018; Glantz et al., 2019). The set of PDEs defines a non-linear reaction-diffusion model dependent on the photoconversion, lipid binding kinetics, and diffusion rates of BcLOV4 (Equation 1). The non-linearity reflects the decrease in available membrane binding sites with protein translocation. The PDEs specifically belong to a category of models termed bulk-surface models, due to the inherent protein coupling between the 3D cytoplasm (the bulk) and 2D membrane (the surface) (Equation 2). This maturing class of models is of paramount importance to studying cell signaling that occurs at the inner leaflet (Madzvamuse and Chung, 2016; Cusseddu et al., 2019; Paquin-Lefebvre et al., 2020).

Bulk-surface FEM on stationary volumes has been described (Madzvamuse and Chung, 2016; Cusseddu et al., 2019; Paquin-Lefebvre et al., 2020). Here, we adapt the model equations to describe the given bulk-surface reaction-diffusion model for a unique 3D volume. Consider the cytoplasm as a volume Ω in $R^{3}$ enclosed by the plasma membrane, a closed hypersurface dΩ. We define $u_{C}\left(x,t\right),v_{C}\left(x,t\right):\text{Ω}\to R^{3}$ as the cytosolic lit and dark-state protein, respectively, and similarly define $u_{M}\left(x,t\right),v_{M}\left(x,t\right):d\text{Ω}\to R^{2}$ as the membrane-bound lit and dark-state protein, respectively; here, the units are molecules/μm^3^ for $u_{C},\;v_{C}\;$, and molecules/μm^2^ for $u_{M},\;v_{M}\;$. We then pose the system of coupled PDEs:

(Equation 1) $$\begin{array}{l} \frac{du_{C}}{dt}=D_{cyto}\nabla^{2}u_{C}+k_{on,p}v_{C}-k_{off,p}u_{C}\\ \frac{dv_{C}}{dt}=D_{cyto}\nabla^{2}v_{C}-k_{on,p}v_{C}+k_{off,p}u_{C}\\ \frac{du_{M}}{dt}=D_{mem}\Delta_{\;d\Omega }u_{M}+k_{on,p}v_{M}-\left(k_{off,p}+k_{off,lit}\right)u_{M}+k_{on,lit}\left(S_{max}-u_{M}-v_{M}\right)u_{C}\\ \frac{dv_{M}}{dt}=D_{mem}\Delta_{\;d\Omega }v_{M}+k_{off,p}u_{M}-\left(k_{on,p}+k_{off,dark}\right)v_{M}+k_{on,dark}\left(S_{max}-u_{M}-v_{M}\right)v_{C}, \end{array}$$

with the bulk-surface coupling enforced using Robin-type boundary conditions (Cusseddu et al., 2019):

(Equation 2) $$\begin{array}{l} D_{cyto}\left(n\cdot \nabla u_{C}\right)=k_{off,lit}u_{M}-k_{on,lit}\left(S_{max}-u_{M}-v_{M}\right)u_{C}\\ D_{cyto}\left(n\cdot \nabla v_{C}\right)=k_{off,dark}v_{M}-k_{on,dark}\left(S_{max}-u_{M}-v_{M}\right)v_{C}, \end{array}$$

where $\Delta_{d\text{Ω}}$ is the Laplace-Beltrami operator, n is the outward-facing unit normal vector to Ω, *D*_*cyto*_ (μm^2^/s) is the cytoplasmic diffusion coefficient, *D*_*mem*_ (μm^2^/s) is the lateral diffusion coefficient along the membrane, *k*_*on*,*p*_ (s^−1^) is the rate of photoconversion from dark to light state, *k*_*off*,*p*_ (s^−1^) is the rate of thermal reversion from lit to dark state, *k*_*off*, *dark*_ (s^−1^) is the rate of unbinding from the membrane in the dark state, *k*_*off*,*lit*_ (s^−1^) is the rate of unbinding from the membrane in the lit state, *k*_*on*,*dark*_ (M^−1^ s^−1^) is the rate of binding to the membrane in the dark state, *k*_*on*,*lit*_ (M^−1^ s^−1^) is the rate of binding to the membrane in the lit state, and *S*_*max*_ (molecules/μm^2^) is the maximal surface density of binding sites. Importantly, all constants are empirically measured in this work (Figure 2B), as detailed later.

Given the non-linearities in the PDEs, model initial conditions were expressed in absolute protein concentration units rather than relative fluorescence units, to allow for direct correlation to single-cell data and inter-cell comparisons. The conversion between mCherry fluorescence to BcLOV4 concentration was calibrated by established methods with intracellular access via patch micropipettes (Cherkas et al., 2018). The cytosolic concentration was typically ∼0.5–3 μM protein (mean = 4.02 μM, 95% confidence interval [CI] = 3.44–4.82 μM, N = 107 cells). The mean of the calibrated single-cell imaging distribution mirrored the population average estimated directly from cell lysate (mean = 3.72 ± 0.79 μM, N = 4 plates) (Figure S1).

The requisite bulk-surface coupling cannot be accurately handled by built-in MATLAB routines or the standard versions of many multiphysics packages. Thus, we derived the weak form and constructed FEM matrices via FELICITY (Walker, 2018). A custom solver (see STAR Methods) was built for the purpose of computational efficiency. Run times ranged from 5 to 10 min per cell (per single core of a 3.7 GHz processor) for a typical experiment (duration = 200 s, Δt = 0.1 s, and maximum element size = 5 μm^3^). Under these 3D mesh conditions, the error was <1% versus the outputs simulated with ultrafine mesh conditions of millisecond resolution on the 10-nm scale for a typical cell (Figure S2). This throughput facilitated the simulation of every experimental cell for the high-resolution studies herein.

### Measurement of biophysical constants

We experimentally derived all constants (Figure 2B) necessary to validate the model and gauge its accuracy. The photoinduced reaction-diffusion model constants capture the flavin photochemistry, diffusion, and membrane binding kinetics. All parameters were measured *in situ* in HEK cells, except for photochemical constants measured by *in vitro* spectroscopy using purified recombinant protein that lacked the mCherry tag.

Diffusion constants were measured by fluorescence recovery after photobleaching (FRAP) microscopy (Figure S3). The measured cytosolic diffusion (*D*_*cyto*_ = 8.932 μm^2^/s) is consistent with our previous estimate (Glantz et al., 2018). The membrane lateral diffusion (*D*_*mem*_ ≤ 0.028 μm^2^/s) is slower than those of proteins bound to a freely mobile lipid or lipid-modified binding partner (0.1–1 μm^2^/s), but unsurprising for a protein that binds the membrane via distributed electrostatic interactions (Kenworthy et al., 2004; Hammond et al., 2009; Ambroggio et al., 2010; Ribrault et al., 2011; Yasui et al., 2014; Bendezú et al., 2015; Natwick and Collins, 2021). The low *D*_*mem*_ indicates that BcLOV4 primarily spreads along the membrane not by lateral diffusion but rather unbinding and rebinding (or “hopping” [Yasui et al., 2014]) events that cause spreading via cytosolic diffusion.

Membrane binding affinity and kinetics of BcLOV4 to the inner leaflet were measured *in cellulo*. The lit-state affinity was *K*_*D*,*lit*_ = 1.19 μM (N = 23, Figures S4A–S4D), which is in line with similarly *in situ*-measured membrane affinities for other anionic lipid binding domains (Smith et al., 2015). The affinity in the dark-adapted state was *K*_*D*,*dark*_ > 20 μM (Figures S4E–S4G), which is reported as a minimum bound due to the lack of observable dark-state membrane localization such that *k*_*on*,*dark*_ could not be determined by our bulk (versus single-molecule) technique in live cells. The maximal surface density (*S*_*max*_) of BcLOV4, which governs its achievable dynamic range and the (non-)linearity of its membrane recruitment, was measured by monitoring maximal achievable cytosolic depletion; the membrane density is calculated from the protein that leaves the cytoplasm given the known surface area-to-volume ratio from the 3D-reconstructed confocal stacks. The measured value of ∼6,000 molecules/μm^2^ (N = 83) is similar to ones measured *in situ* for overexpressed proteins that bind endogenous membrane lipids (Smith et al., 2015).

### PSF convolution for comparing models with real-world data

Finally, we convolved the model output with the optical hardware PSF to improve direct comparison of model outputs to microscopy findings. Likewise, the optical input of the framework also factors in the diffraction-limited excitation beam profile to account for off-focal plane excitation, which can impact the signaling induced by cytosolically diffusible proteins.

Owing to diffractive effects, observed membrane fluorescence is inherently a partial function of cytosolic protein concentration, where the ∼7-nm-thick membrane accounts for just a fraction of the ∼100–400 nm axial depth-of-field of optical microscopes at high magnification. Thus, measurements of dynamic localization that equate membrane density with membrane fluorescence will vary widely with the hardware system. (Figures S5A and S5B). However, fluorescence quantification of the larger cytoplasm can be less susceptible to these confounds because its fluorescence is not as affected by diffractive effects or subtle morphological changes (Figure S5C). Hence from here on, measurements of protein depletion and recovery are done by tracking cytoplasmic fluorescence.

### Single-cell model performance

**Figure 3:**
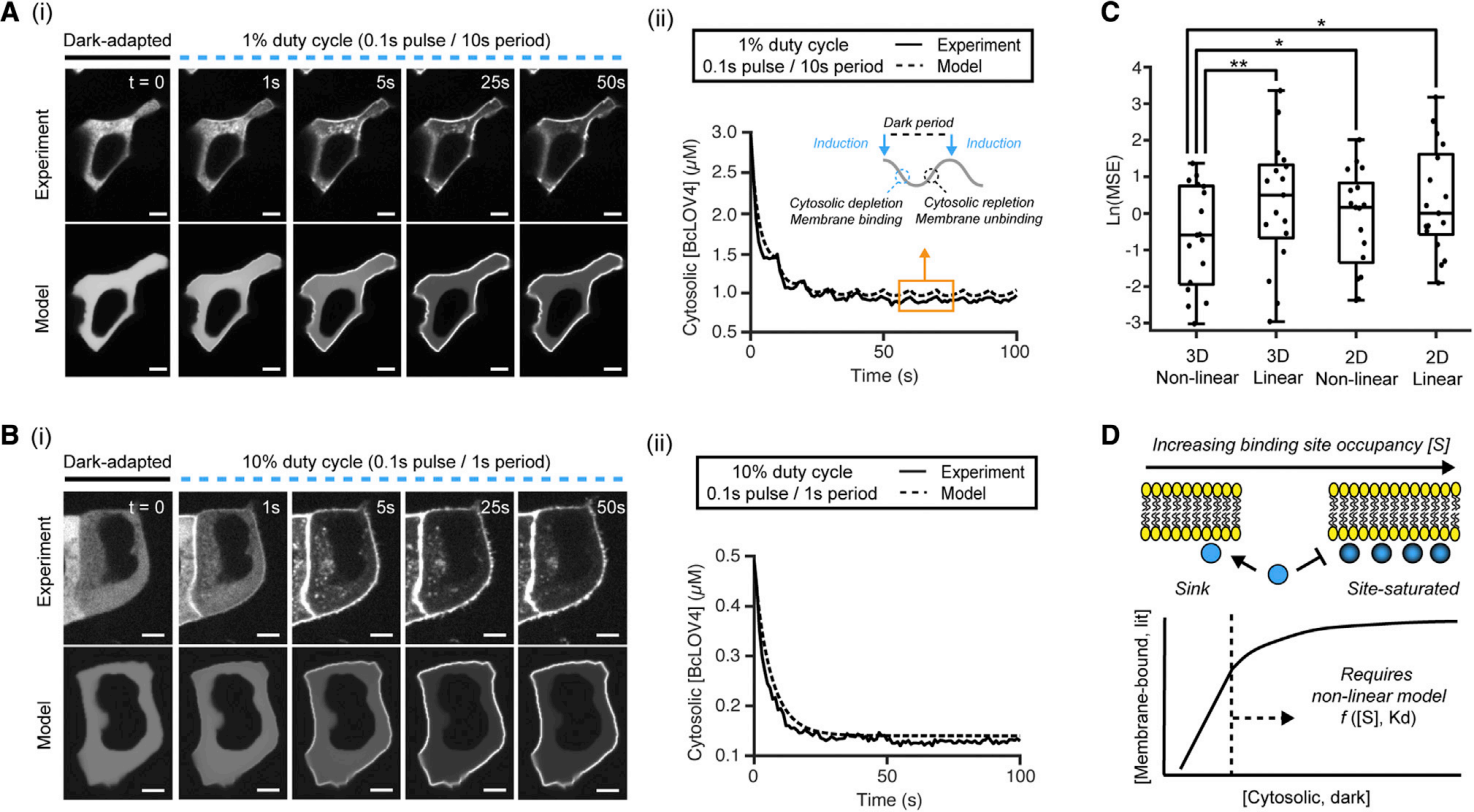
Single-cell finite element models for pulsatile whole-field (unpatterned) stimulation with confocal imaging BcLOV4 refers to BcLOV4-mCherry. Predicted model outputs closely mirror experimental data across duty cycles (φ; 0.1 s blue light pulses, 12.24 W/cm^2^). Cytosolic depletion and recovery are quantified in lieu of membrane fluorescence that is more susceptible to confounds from diffraction and cell motility (Figure S5). (A) φ = 1%. (i) Experiment and corresponding model prediction. (ii) Cytosolic depletion time course of same cell; inset schematizes the phases of a stimulatory period. Scale bar, 5 μm. (B) φ = 10%. (i) Experiment and model. (ii) Cytosolic depletion time course of same cell, which lacks perceivable membrane unbinding or cytosolic repletion during each 1 s period. Model recapitulates nuclear void and subcellular distribution in the cytosol and plasma membrane but does not account for neighboring cells or for lysosome binding that greatly increases computational time with minimal accuracy benefit (Figure S6). Scale bar, 5 μm. (C) Mean-squared error (MSE) of the described 3D non-linear model (of panels A and B) versus a 3D linear model, 2D non-linear model, and 2D linear model. Pooled dataset contains cells stimulated at φ = 0.67%–10% (N = 17). Linear models performed worse for high protein concentrations (Figures S2D–S2F). Paired Wilcoxon signed rank test: ∗p < 0.05, ∗∗p < 0.01. (D) Schematized non-linear modeling of dynamic membrane recruitment. The system is linear at low membrane binding site occupancy or low cytosolic BcLOV4. High concentrations typical of overexpressed inducible/optogenetic signaling systems require non-linear models due to high fractional occupancy of membrane binding sites upon photoactivation.

We compared model outputs with results from spinning-disk confocal stimulation and imaging of BcLOV4 (Figure 3). The model was able to mirror the salient spatiotemporal behavior of BcLOV4, including undulations in cytosolic protein level from repetitive unbinding/rebinding cycles in response to sparsely pulsatile stimulation (Figures 3A and 3B). Deconvolution of confocal z-stack data before geometry generation did not significantly impact resultant cell meshes or cell-wide mean-squared error, likely due to the excellent axial sectioning capabilities of confocal microscopy.

Like any other computational approach, the model’s accuracy depends on the granularity of the initial mesh conditions. For example, the standard output does not account for shared membranes with neighboring cells or for lysosomal voids that appear as fluorescent puncta upon post-induction protein binding (Figures 3A and 3B). Accounting for lysosomal binding had limited impact on BcLOV4 membrane recruitment accuracy at normal biogenesis levels, but slowed the simulation by ∼10-fold, scaling by O(n^2^) the number of lysosomes (Figure S6).

The 3D non-linear modeling approach was more accurate than 3D linear and 2D (non-)linear modeling (Figures 3C and S2D–S2F; see STAR Methods for calculation of error). Note that this comparison represents a best-case scenario for the 3D linear and 2D (non-)linear models, since we initialized them with the absolute protein concentrations rather than with relative or assumed concentrations (Valon et al., 2015; Natwick and Collins, 2021). These data suggest that both the geometric aspects and the non-linearity contribute to model performance. They also suggest that linear models of membrane recruitment that treat the membrane as an infinite binding sink (Figure 3D), while valid at low cytosolic concentrations, are less appropriate for overexpressed proteins in engineered cells. This influence can specifically be seen by the poor performance of linear models for high-concentration cells (Figure S2F).

### Intrinsic and extrinsic determinants of spatial confinement

**Figure 4:**
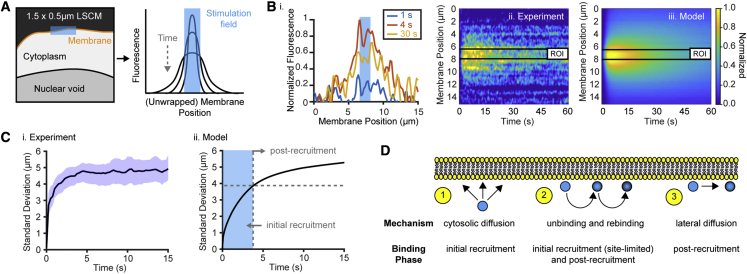
Spatial confinement of BcLOV4 BcLOV4 refers to BcLOV4-mCherry. (A) Scheme of laser-scanning confocal microscopy (LSCM) patterning of a narrow peri-membrane excitation ROI (∼1.5 × ∼0.5 μm, blue region, λ = 405 nm). Confinement measurements tracked fluorescence time course along the membrane profile (orange line). (B) Membrane profile fluorescence evolution of a representative cell. (i) Unwrapped profile (blue shaded area denotes excitation region). (ii) Heatmap and (iii) corresponding model prediction of complex spatiotemporal dynamics generally agreed. Black lines denote excitation region. Heatmaps are normalized so that peak fluorescence over the entire time course is set to 1. (C) SD of the Gaussian profile (solid black line ± 95% CI, N = 14) from the heatmaps, a proxy for the degree of spatial confinement. (i) Experiment and (ii) model prediction. The SD initially rapidly increases because of cytosolic diffusion-limited association. BcLOV4 is then largely immobile due to slow lateral diffusion and membrane affinity. (D) Biophysical processes (i.e., excluding hardware contributions) that govern spatial resolution of optically inducible recruitment, pre-/post-binding to the membrane. See Figure 5 for sensitivity analysis.

We next used our single-cell approach to explore how optical stimulation paradigm and hardware affect spatiotemporal resolution, specifically the spatial confinement of membrane recruitment, a motivating metric in optogenetics. In brief, a small 1.5 × 0.5 μm stimulatory region of interest (ROI) was patterned by laser-scanning confocal microscopy (LSCM), and the resultant fluorescence membrane profile over 1 min was fit with a Gaussian to estimate the membrane spread (Figures 4A–4C). The spatial spread primarily occurs during the initial few seconds post excitation when the protein rapidly diffuses in the cytoplasm (Figures 4C and 4D, N = 14 cells). Upon binding, the spreading occurs by lateral diffusion and by rebinding events subject to cytosolic diffusion. While great attention in optogenetics has been given to minimizing lateral diffusion to reduce spreading (Valon et al., 2015; Van Geel et al., 2018), its contributions to spatial resolution are likely outweighed by cytosolic diffusion when the photoactivated protein is cytosolic, as is commonly the case.

**Figure 5:**
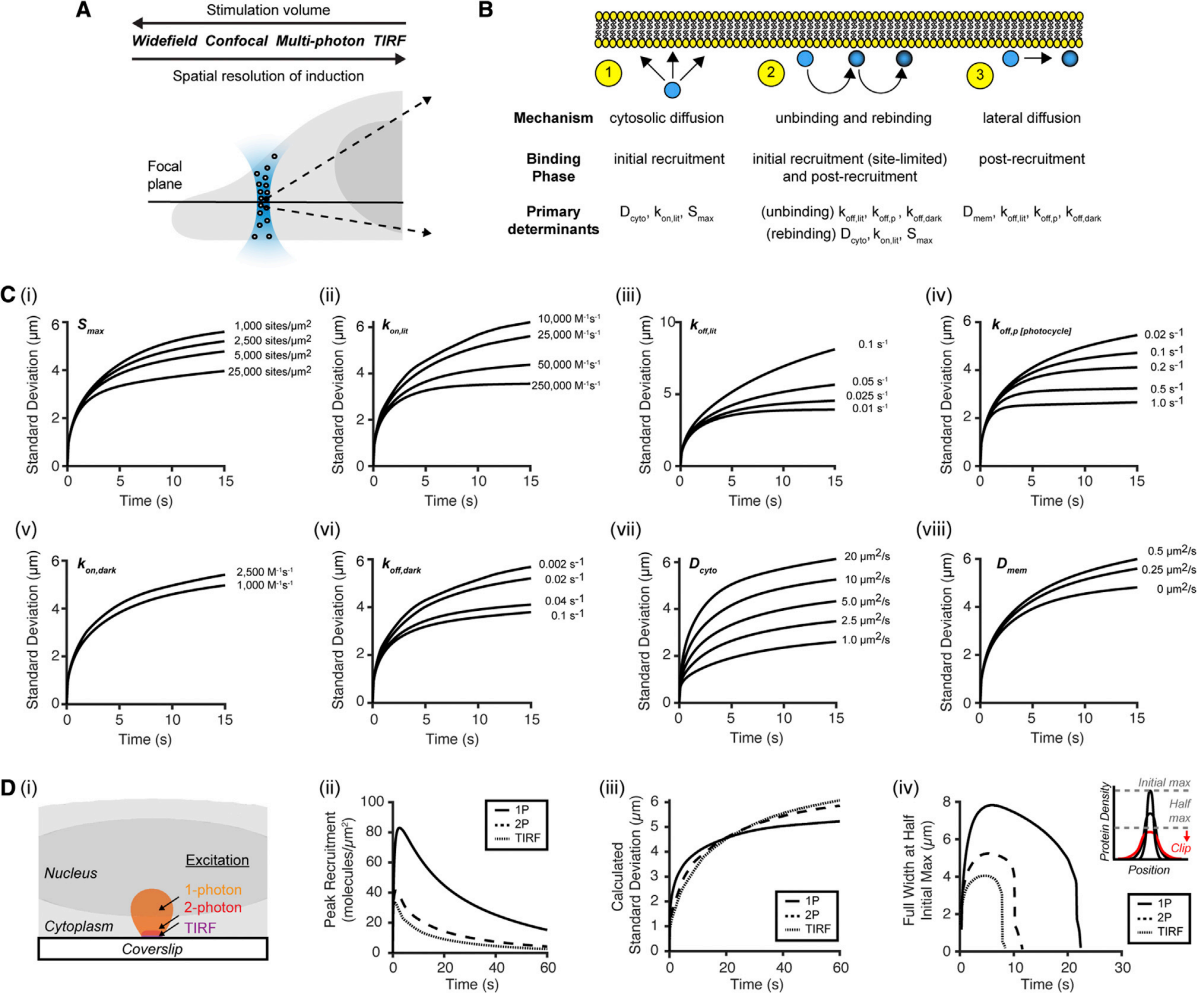
Determinants of spatiotemporal resolution and signaling magnitude of optically inducible membrane recruitment BcLOV4 refers to BcLOV4-mCherry. (A) Diffractive differences between hardware for induction. Larger stimulation volumes increase cytosolic diffusion lengths and consequently decrease spatial resolution. (B) Spatial confinement post stimulation impacted by: (1) cytosolic diffusion distance before membrane binding; (2) unbinding frequency and distance traveled before rebinding, and (3) distance traveled by lateral diffusion. (C) Sensitivity analysis of spatial confinement to the intrinsic biophysical parameters. Values span those derived here for BcLOV4 and elsewhere for heterodimerization systems. SD of the protein distribution along a modeled membrane matching the experimental conditions of Figure 4. (i) Binding site availability (S_max_), (ii) k_on,lit_, (iii) k_off,lit_, (iv) k_off,p_ of the chromophore photocycle, (v) k_on,dark_, (vi) k_off, dark_, (vii) cytosolic diffusivity, or (viii) lateral diffusivity along the membrane. The role of lateral diffusion is limited for large excitation volumes and/or when binding kinetics permit extensive cytosolic diffusion when rebinding. (D) Effect of stimulation method. Systems with larger excitation volumes drive more potent signaling but reduce spatial precision. (i) Simulated excitation of 2 × 2 μm region at the bottom of a model cell (excitation duration = 100 ms, cytoplasmic [BcLOV4] = 1 μM) by different stimulation methods (10 W/cm^2^ irradiance at focal plane). Highlighted volumes show the 1 W/cm^2^ isosurfaces of the excitation volume. (ii) Peak membrane-bound protein recruitment, baselined to subtract pre-bound protein in the dark state. The rapid drop-off in peak recruitment for axially confined stimulation (2P, TIRF) due to low levels of activated cytosolic BcLOV4 results in decreased induced signaling. (iii) Spatial resolution quantified by SD for a Gaussian distribution fit (1P) or the full width at half maximum /2sqrt(2ln(2)) (2P, TIRF) of the membrane profile at each time point. Axially confined methods do not outperform classic 1P excitation for this measure overall, but better retain focality during initial recruitment (t < 5–10 s). (iv) Spatial resolution quantified by width at half of initial/absolute maximum. Inset schematizes the steep drop-off at longer time points by this metric from threshold clipping.

To systematically explore the determinants of spatial confinement, we simulated both the molecular-level biophysical interactions and the optical hardware used for induction and visualization (Figures 5A and 5B). We predicted the sensitivity of spatial confinement to these two parameter classes for a typical protein concentration of 1 μM (Figures 5C and 5D). For the biophysical parameter sensitivity analysis, we used the cellular geometry from the confocal experiment of Figure 4 and a range of biophysical constants informed by experiments here or elsewhere (Bartelt et al., 2018; Natwick and Collins, 2021). For hardware analysis, we used a geometry generated by hemi-ellipsoid projection of an idealized cell (see STAR Methods).

Spatial resolution was most profoundly improved by parameters (lower *D*_*cyto*_, higher *k*_*on*,*lit*_, lower *k*_*off*,*lit*_, and higher *k*_*off*,*p*_) that directly decrease the average distance traveled by a cytosolically diffusing lit-state protein by slowing it, improving binding, decreasing rebinding, or decreasing the lit-state lifetime (Figure 5C). An ideal *D*_*mem*_ = 0 can only improve the confinement to the spatial resolution limit set by the other parameters (Figure 5Ci–viii). However, *D*_*mem*_ is indeed limiting for the less common heterodimerization configuration where the photosensory protein is the membrane-localized component (Toettcher et al., 2011; Natwick and Collins, 2021). Of note, increasing *S*_*max*_ improves resolution by reducing diffusion times and distances; it also enhances the maximum possible inducible signaling (Natwick and Collins, 2021).

The analysis of how optical hardware excitation volume impacts recruitment spatiotemporal dynamics found that a laser-scanning confocal system (1P) induces greater signaling than more axially precise total internal reflection fluorescence (TIRF) and 2P, by exciting a proportionally larger volume for similar membrane surface areas (Figure 5D). When only one optical pulse is delivered, TIRF and 2P potentially become less focal, with increasing SD as membrane-bound protein continues to spread after the excitation volume depletes of photoactivated protein (greater than tens of seconds); conversely in 1P, cytosolic photoexcited proteins continue to populate the illuminated surface area. If confinement is defined as the profile width at half the experimental maximum (instead of instantaneous relative maximum), TIRF and 2P are more focal because the protein densities are rapidly clipped by the quantification threshold. Thus, the primary spatiotemporal benefit of axial confined TIRF and 2P is an improved impulse response of induced signaling.

### *In silico* resolution of inter-experiment variability

**Figure 6:**
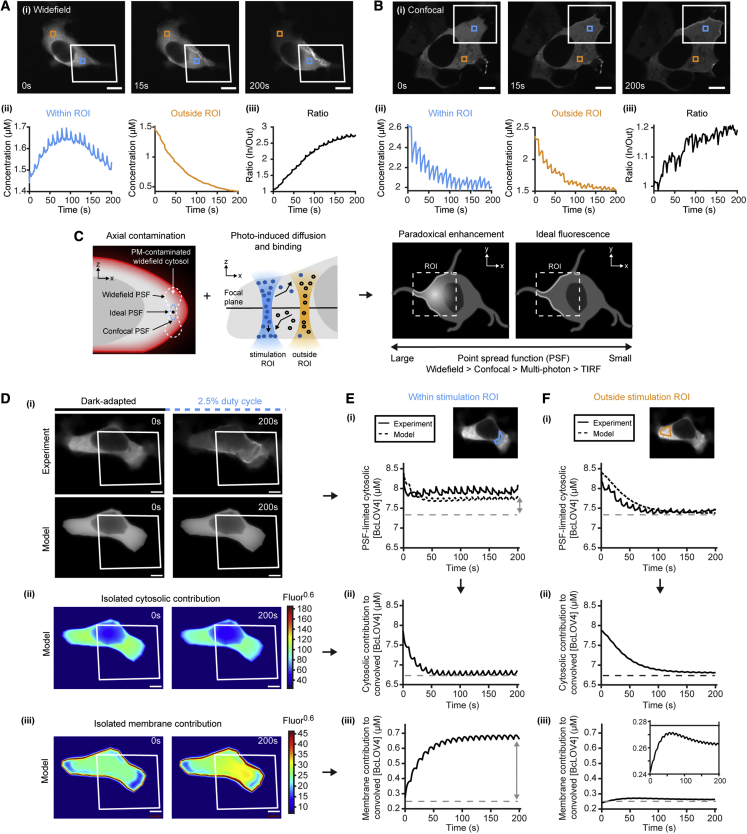
3D FEA-derived resolution of hardware-dependent interpretive confounds BcLOV4 refers to BcLOV4-mCherry. (A) Example of observed paradoxical fluorescence enhancement. Widefield imaging of DMD-excited cells (12 mW/cm^2^, duty cycle φ = 10%) shows (i) apparent protein depletion from distal unstimulated regions and notable brightening within the stimulation field (white box). Scale bar, 10 μm. (ii) Fluorescence-derived cytosolic concentration traces within stimulation field (cyan quantification box in i), distal unstimulated region (orange box in i), and their (iii) ratio normalized to 1. The fluorescence enhancement in the stimulation field erroneously suggests photoinduced diffusional gradients lead to protein accumulation within the stimulation field. (B) Confocal images of DMD-excited cells do not show the large fluorescence enhancement within the stimulation region. Analyzed as in (A). (C) Model-derived explanation of paradoxical cytosolic fluorescence enhancement within a stimulation field by PSF-dependent axial signal integration of membrane-bound protein. The cytosol darkens quickly outside the field whereas the brightening within it counteracts photobleaching. See (D), (E), and (F) for decomposition. (D) Model of single cell with approximated contours by volumetric extrapolation of one focal plane. (i) Cell and corresponding simulated image in response to patterned illumination (red box, 12 mW/cm^2^, φ = 2.5%). Geometric uncertainty of the initial mesh precludes pixel-wise accuracy of the output. Gamma corrected (γ = 0.6) to improve discrimination. Scale bar, 5 μm. (ii) Decomposition of the model output. Theoretical isolated contribution of cytoplasmic protein to the model result in (i). (iii) Theoretical isolated contribution of membrane-bound protein to the model result in (i). (E and F) Partial recapitulation of rebinding phenomena observed (E) within and (F) outside the stimulation field. (i) PSF-limited cytosolic depletion time course (photobleach-corrected). Inset: simulated initial image with measurement field overlaid. (ii) Theoretical cytosolic and (iii) membrane fluorescence contributions to the net signal. The ∼0.4 μM equivalent difference between the two cytosolic regions (E-i and F-i) cannot be explained by cytosolic concentration (E-ii and F-ii), but can be by PSF-limited integrated fluorescence of membrane-bound protein (E-iii and F-iii). Dotted lines denote approximate steady-state level outside the stimulation field.

Given its accuracy in predicting cell-wide dynamics, the framework provides a powerful means to improving robustness in single-cell studies by bridging inter-experiment variability *in silico*. To this end, we explored differences between subcellular digital micromirror device (DMD)-stimulation experiments on widefield versus confocal microscopes. We observe a phenomenon on widefield microscopes whereby cytoplasmic fluorescence appears enhanced within a patterned stimulation ROI, as if it continuously recruits protein from distal unstimulated regions (Figure 6A). The paradoxical enhancement is the opposite of the expected rapid cytoplasmic depletion from the ROI. It is not observed when we use a confocal microscope to image a similarly DMD-stimulated cell (Figure 6B). This discrepancy suggests that the enhancement is due to the poor axial sectioning capability of a widefield microscope (Figure 6C). Post hoc analysis found that the hardware-dependent imaging artifact stems from the combination of (1) PSF-limited photonic integration of membrane-bound protein within the ROI and (2) simultaneous photobleaching throughout the cell (Figures 6D–6F).

**Figure 7:**
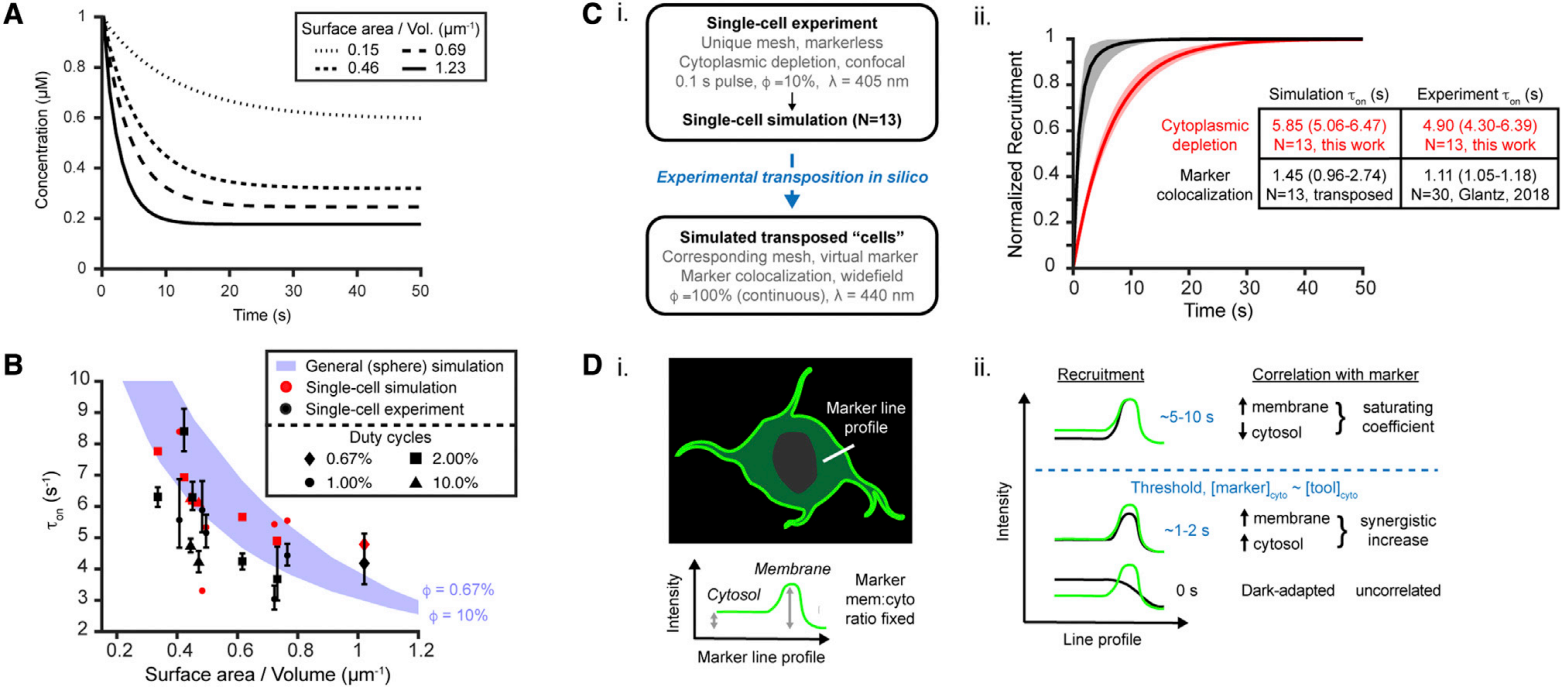
Computational resolution of inter-experimental differences in observed membrane association kinetics (τ_on_) BcLOV4 refers to BcLOV4-mCherry. Kinetics measured in one experiment can be reasonably extrapolated to data generated in another (e.g., different cell or experimental condition). (A) τ_on_ varies across a physiological range of cell surface area-to-volume ratios (SA/V) that determine membrane binding site availability relative to total intracellular BcLOV4 (0.1 s pulse, λ = 405 nm, duty cycle φ = 10%, 12.24 W/cm^2^). SA/V range of ∼0.15–1.2 mm^−1^ spans geometries from a large 20-μm-radius cell with negligible nuclear fractional volume to a small 5-μm-radius cell with sizable 4-μm-radius nucleus. (B) Log-order acceleration of τ_on_ with increasing SA/V as the main contributor to intercellular differences in recruitment kinetics. Experimental cells and corresponding FEM (experiment: black, 95% CI; simulation: red) and calculated τ_on_ (blue band, in idealized spherical cells) across SA/V and stimulation duty ratios (as in A, φ = 0.67%–10%). (C) *In silico* “transposition” of a cell between experiments. (i) Validated cell-unique FEM from a pulsatile stimulation experiment with τ_on_ quantified by cytosolic depletion is simulated as if it was in a different experiment of continuous stimulation and quantified by colocalization with a virtually introduced membrane marker. (ii) Simulated transposition of one cell with virtual GFP-CAAX (experiment-derived marker background fluorescence of ∼10% relative to its membrane fluorescence; error = 95% CI; ∼0.5 μM protein to match previous work [Glantz et al., 2018]). Colocalization correlation analysis along a line profile transecting the membrane results in faster perceived recruitment dynamics than by cytoplasmic depletion. Predictions on markerless experimental cells using virtual markers here agree with previous work (inset table). (D) Post hoc analysis reconciles data quantified by the two methods. (i) Schematized GFP-CAAX marker with constant membrane/cytosol ratio. (ii) Tool∷marker correlation initially improves in both the membrane and cytosol to synergistically accelerate perceived colocalization increase. The correlation improvement subsequently slows when membrane colocalization increase is counterbalanced by cytosolic colocalization decrease (when cytosolic BcLOV4 depletes beyond the cytosolic marker level).

Next, we explored how cell geometry affects the observed kinetic constant for membrane association (τ_on_), a common metric for comparing optogenetic recruitment tools (Buckley et al., 2016; Zimmerman et al., 2016). While it is known that cell size and geometry can impact signaling (McBeath et al., 2004; Meyers et al., 2006; Neves et al., 2008; Hara and Kimura, 2009), these factors are often overlooked in optogenetics. Specifically, the plasma membrane surface area to cytoplasmic volume ratio (SA/V) critically impacts the binding site availability *S*_*max*_ relative to total number of diffusible molecules. Increasing SA/V accelerates τ_on_ by minimizing the roles of hopping and non-linearity, as observed in single cells across heterogeneous protein concentrations and stimulation duty ratios (Figures 7A and 7B, N = 13).

We also demonstrated that a cell-unique model experimentally validated in one experiment can be used to predict the outcome of another of entirely different hardware, stimulation paradigm, and analysis (Figure 7C). We compared τ_on_ determined by (1) fitting cytosolic depletion from confocal micrographs of pulsatile-stimulated cells (i.e., method used here) versus (2) colocalization correlation analysis with a membrane marker from widefield images of continuously stimulated cells (our previous method [Glantz et al., 2018]). Notably, the marker here is purely virtual, not coexpressed as before. *In silico* transposition recapitulated a salient interpretive confound that τ_on_ derived by cytoplasmic depletion (∼5 s) is systematically slower than by marker colocalization (∼1.5 s). The latter perceived acceleration is a consequence of the mathematical correlation (Figure 7D). Because the marker has background cytoplasmic fluorescence, the acceleration reflects the synergistic increase in correlation along both the cytosolic and membrane segments and a faster approach to mathematical saturation. Such predictive simulations show how computational approaches can improve interpretive robustness by permitting results from one lab or experiment to be compared with those emulating another.

### Implementation in other computational environments

While the mesh generator and solver were custom-built for computational efficiency, the single-cell framework can be implemented in other software. It is modular in that the (1) 3D cellular mesh, (2) time-varying 3D-patterned distribution of optical excitation, (3) reaction-diffusion model (or other relevant model), and (4) PSF convolution can all be treated as separate components imported and exported between computational environments. Thus, we implemented the approach in VCell (Figure S7), a general environment for simulating cell signaling (Moraru et al., 2008) that can natively mesh a 3D cell (based on the finite volume method, with a uniform orthogonal mesh) and account for PSF convolution. We integrated the calculation of spatiotemporally complex 3D distributions of optical excitation (e.g., laser raster) into VCell by introducing the optical input distribution computed within our framework as a specific “light” element, taking inspiration from work elsewhere in modeling FRAP experiments (Kühn et al., 2011) (Figure S7A).

We successfully replicated our analyses in an end-to-end fashion with closely approximated results (Figures S7B–S7D, related to Figure 5D). The computational runtime increased in VCell versus the fully custom solution (∼10-fold slowdown to simulate a TIRF experiment). Since its inputs are continuous functions, VCell recomputes the intensity of excitation light at each meshpoint at every timestep, even when it is unchanged. Conversely, the custom FEM can utilize coarser mesh sizes to reconstruct the topologies and store the optical input elements in local memory. Nevertheless, the successful replication demonstrates that one can employ the modeling tools of choice and those best suited for the single-cell modeling task at hand.

## Discussion

Predictions by our single-cell framework agreed with experimental single-cell data. Many features contribute to its utility in studying peripheral membrane protein dynamics: 3D non-linear dynamics without concentration assumptions, volumetric reconstructions of single cells, optical beam profiling and PSF convolution to simulate hardware-specific effects, and validation by a global optimal solution for the set of PDEs (i.e., all the fundamental constants and absolute concentrations were measured).

While we successfully simulated anticipated outcomes and visual outputs of single-cell experiments, the quality of the simulated micrograph depended (1) on the mesh fidelity of the initial volumetric reconstruction as already described, and (2) on the underlying biophysics accounted for in the model. For example, the model does not recapitulate BcLOV4 recruitment “hot spots” (e.g., punctate membrane fluorescence in Figures 1 and 3A), which likely form by *de novo* aggregation on the membrane given that BcLOV4 is monodisperse in the cytosol (based on identical FRAP-measured and theoretical cytosolic diffusivities). While we cannot exclude a role for curvature in this phenomenon, our current understanding from *in vitro* studies (of lipid headgroup selectivity) of the BcLOV4 membrane interaction is that it is more governed by membrane anionic charge density than by curvature (Glantz et al., 2018).

### Optogenetic membrane recruitment-based signaling implications

Single-component BcLOV4 recruitment reaches a fractional occupancy of [*S*]*/S*_*max*_ ∼ 30% for typical cellular concentrations, similar to the occupancy reached by the iLID system (Natwick and Collins, 2021). Since the total induced signaling magnitude depends on the cytosolic concentration of the payload, a single-component system whose surface-bound partner is in natural excess can decrease the metabolic load by ∼80% over an optimized heterodimerization system, i.e., one expressed with a 4-fold excess of the surface component (Natwick and Collins, 2021). Likewise, since the BcLOV4 *S*_*max*_ (∼6,000 molecules/μm^2^) exceeds that of lipidated peripheral membrane proteins (500–1,500 molecules/μm^2^ [Del Piccolo and Hristova, 2017; Angert et al., 2020]), BcLOV4 likely has a greater achievable membrane density than a heterodimerizer; consequently, it may drive greater signaling induction over a broader concentration range of linear binding kinetics.

It was unanticipated that when the photosensory domain is cytosolic, its cytosolic diffusion has a much larger role than lateral diffusion in the spatial confinement, which becomes especially important under binding-limiting cases. Also unexpectedly, we found that axially confined stimulation improved the impulse response of induced signaling more than the spatial resolution. However, in systems with a membrane-localized photosensor, the confinement should be determined primarily by lateral diffusion and is largely independent of excitation volume. Thus, a system with a membrane-localized photosensor is likely more ideal for maximizing spatial confinement; however, this configuration comes at the expense of increased metabolic load and/or decreased signaling magnitude for the fractional occupancy reasons described above.

Our studies elucidated the biophysical determinants of performance in optogenetic membrane recruitment and emphasized how *in silico* approaches can enhance interpretive robustness in single-cell signaling studies. The validated framework provides a foundation for the principled design of improved tools and complex stimulation paradigms for tight and potentially closed-loop spatiotemporal control of cell signaling. Considerable progress in optogenetics has been made in model-guided signaling gradient formation (de Beco et al., 2018; Izquierdo et al., 2018), and improved strategies could further advance the field in the study of oscillatory waves and more complex pattern formation (Chiou et al., 2018; Hörning and Shibata, 2019; Borgqvist et al., 2021).

### FEM in cell signaling

FEM is widely used to study mechanics and transport, including in cell motility (Neilson et al., 2010; Elliott et al., 2012) and deformation biomechanics using non-stationary surface elements (Boccaccio et al., 2011; Hughes et al., 2018). Its application for spatially modeling intracellular signaling is less common but more theoretically advanced for describing a coupled bulk-surface system of membrane recruitment than alternative approaches (Madzvamuse and Chung, 2016; Cusseddu et al., 2019; Paquin-Lefebvre et al., 2020). Beyond equation-based applications, rules-driven or agent-based modeling frameworks such as PhysiBoSS (Letort et al., 2019) are particularly useful when spanning multiple scales, such as incorporating intracellular signaling cascades with cell-cell interactions (Bonabeau, 2002; Gorochowski, 2016). Most existing multi-scale agent-based frameworks of cell signaling still approximate cells as ideal spheres. We envision that single-cell FEM and excitation light beam computation techniques described herein are extendable to rules-driven models and would benefit them by facilitating tolerance to experimental imaging conditions and cell heterogeneity.

In summary, we described a conceptual framework that can improve computational models of single-cell signaling and microscopy experiments. We applied it to study BcLOV4 spatiotemporal dynamics to identify key performance determinants in optogenetic membrane recruitment-based signaling. We demonstrated how the approach can be used for improved robustness when interpreting single-cell data. By accounting for both the intrinsic biophysical contributions of the optogenetic tool and the extrinsic contributions of the optical system used for stimulation and imaging, this approach can guide forward the design of improved optogenetic tools and experiments for dissecting peripheral membrane protein signaling dynamics.

### Limitations of the study

The current framework was designed for single-machine computation. Future work includes expanding its throughput via distributed computation functionality. While VCell models can be distributed to run on its cloud server, the current study was not compatible with this online server owing to the presence of spatiotemporally varying inputs. Specifically, VCell does not natively provide a simple interface through which to model time- and space-varying inputs at this time. The described local VCell implementation introduced this functionality without access to the underlying codebase; this approach required single-machine computation. Accordingly, the described runtime performance assessments represent hardware-matched head-to-head comparisons. Despite these limitations, the described framework is more broadly envisioned as an adjunct to existing tools and is intended to streamline the analysis of complex single-cell signaling studies.

## STAR★Methods

### Key resources table

**Table:** 

REAGENT or RESOURCE	SOURCE	IDENTIFIER
**Bacterial and virus strains**		

BL21(DE3)	New England Biolabs	C2527H

**Chemicals, peptides, and recombinant proteins**		

Dulbecco’s Modified Eagle’s Medium with GlutaMAX	Invitrogen	10,566,016
Penicillin-Streptomycin	Millipore Sigma	P4333
Fetal bovine serum	Millipore Sigma	F2442
L-α-phosphatidylcholine	Avanti Polar Lipids	840,054
Sulforhodamine 101 (Texas Red)	Millipore Sigma	S7635
Texas Red-DHPE	Biotium	60,027
Fluorescent polystyrene beads	Degradex	2101C/2211
Fluorescent microspheres	Spherotech	FP-0252-2/FCM-02556-2
Lucifer Yellow	Biotium	80,015
TransIT-293 transfection reagent	Mirus Bio	MIR2700
Mg-ATP	Millipore Sigma	A9187
Na-GTP	Millipore Sigma	G8877

**Deposited data**		

Raw data	This work	Mendeley Data: https://doi.org/10.17632/62xbycxn4y.1

**Experimental models: Cell lines**		

HEK293T	ATCC	CRL-3216

**Recombinant DNA**		

BcLOV4_mCherry_pcDNA3.1	Addgene	114,595
His6_BcLOV4_mCherry_BamUK	Addgene	114,596
LAMP1_miRFP670nano_pN1	Addgene	127,435

**Software and algorithms**		

MATLAB	MathWorks	https://www.mathworks.com/products/matlab.html
Fiji	Schindelin et al., 2012	https://fiji.sc/
Python (version 3.8)	Van Rossum and Drake, 2009	https://www.python.org
FELICITY	Walker, 2018	https://github.com/walkersw/felicity-finite-element-toolbox/wiki
The Python Microscopy Environment	Soeller Lab	https://www.python-microscopy.org/
Virtual Cell	Moraru et al., 2008	https://vcell.org
PyOptica	https://pypi.org/project/pyoptica/	https://pyoptica.gitlab.io/pyoptica-blog/
PSF (version 2021.6.6)	https://github.com/cgohlke/psf/	https://pypi.org/project/psf/
Numpy (versions 1.20.0)	Oliphant, 2006	https://numpy.org/
PSF Generator	Kirshner et al., 2013	http://bigwww.epfl.ch/algorithms/psfgenerator/
FEM toolbox	This work	https://github.com/brianchowlab/BcLOV4-FEM (https://doi.org/10.5281/zenodo.6587636)
Analysis scripts	This work	https://github.com/brianchowlab/reproducibility-BcLOV4-FEM (https://doi.org/10.5281/zenodo.6587665)

**Other**		

HisTrap FF	Cytiva	17,525,501
Amicon Ultra-4 centrifugal filter unit	Millipore Sigma	UFC901008D
Quartz cuvette	Starna Cell	16.100F-Q-10/Z15
Power meter	Thorlabs	PM100D
455 nm LED	Mightex Systems	0301
Spectrophotometer	Ocean Insight	USB2000+
Widefield microscope	Leica	DMI6000B
Spinning Disk confocal microscope	Nikon	ECLIPSE Ti2
Spinning disk confocal scanner unit	Yokogawa	CSU-W1
Digital light projector	Digital Light Innovations	CEL5500
Laser-scanning confocal microscope	Leica	TCS SP8

### Resource availability

#### Lead contact

Further information and requests for resources and reagents should be directed to and will be fulfilled by the lead contact, Ivan Kuznetsov (ivan.kuznetsov@pennmedicine.upenn.edu).bchow@seas.upenn.edu

#### Materials availability

This study did not generate new materials. The BcLOV4-mCherry plasmids for bacterial expression (His6_BcLOV4_mCherry_BamUK, ID: 114596) and mammalian expression (BcLOV4-mammalian-recoded_mCherry_pcDNA3.1, ID: 114595) are available from Addgene.

### Experimental model and subject details

#### Cell lines

HEK293T (ATCC, CRL-3216) cells were cultured in D10 media composed of Dulbecco’s Modified Eagle's Medium with GlutaMAX (Invitrogen 10566016), supplemented with Penicillin-Streptomycin at 100 U/mL and 10% heat-inactivated fetal bovine serum (FBS). Cells were maintained in a 5% CO_2_ tissue-culture treated dishes in a water-jacketed incubator (Thermo/Forma 3110) at 37°C.

### Method details

For the following sections, unless otherwise specified: (i) water was Milli-Q water (ddH2O, 18.2 MΩ · cm), (ii) oligonucleotides were synthesized by IDT, (iii) genetic constructs were verified by Sanger sequencing (Genewiz), (iv) all experiments referring to BcLOV4 specifically refer to the BcLOV4-mCherry fusion construct, unless specifically stated otherwise. Native sequence and mammalian codon-optimized BcLOV4 plasmids are publicly available via Addgene.

#### Genetic constructs, protein expression and transfection

##### Recombinant BcLOV4 production

BcLOV4 was bacterially produced and purified by FPLC (fast protein liquid chromatography) as described previously (Glantz et al., 2018). Protein was stored at 4°C and used within 2 days of purification.

##### Photocycle kinetics and quantum efficiency

BcLOV4 (without fused mCherry) absorbance scans were used to determine photocycle kinetics by monitoring absorbance at 450 nm (A450) as previously described (Glantz et al., 2018). To measure quantum efficiency of photoconversion (φ) and *k*_*on*,*p*_, 15 s of baseline measurements were made at room temperature, and the A450 immediately was recorded after blue light stimulation of varying intensity (5 s, λ = 455 nm, 0.06–7.43 mW/cm^2^). To extract the constants, we can model the amount of non-photoactive BcLOV4 (c) as:

(Equation 3) $$\frac{dc}{dt}=-k_{on,p}c$$

where we can ignore lit-to-dark state thermal reversion because it occurs on timescales much longer than photoactivation; the fraction of activated BcLOV4 after 5 s of excitation will be $1-e^{-5k_{on,p}}.$ The constants *k*_*on*,*p*_ and φ are related to each other by the rate of photon absorption:

(Equation 4) $$k_{on,p}=\int \varphi \varphi_{p}\sigma_{ex}d\lambda$$

where $\sigma_{ex}$ is the absorption cross section in units of cm^−2^, $\varphi_{p}\;$ is the photon flux density in units of photons cm^−2^s^−1^, and λ is the wavelength of excitation light. Given:

(Equation 5) σex=2303ϵNA

where ϵ is the molar extinction coefficient (∼12,500 M^−1^s^−1^) and $N_{A}$ is Avogadro’s number, and:

(Equation 6) $$\varphi_{p}=\frac{P_{d}}{h\nu }$$

where $P_{d}$ is the power density of the excitation light, h is Planck’s constant, and ν is the frequency of excitation, we approximate this integral by assuming that our LED spectrum is narrow and centered at 455 nm:

(Equation 7) $$k_{on,p}\approx {\varphi \varphi_{p}\sigma_{ex}|}_{\lambda =455\;nm}$$

The fraction of BcLOV4 that is activated after 5 s is:

(Equation 8) $$f=1-e^{-5\varphi \varphi_{p}\sigma_{ex}}$$

which can be fit to the experimental data to extract φ and then use the value to calculate $k_{on,p}$.

Dark-state reversion kinetics were measured by monitoring the absorbance at 450 nm (A450) post-stimulation for every 0.5 s for 2 min, and *k*_*off*,*p*_ was fit as an exponential to reversion kinetics.

##### Mammalian cell culture and transduction

HEK293T (ATCC, CRL-3216) cells were cultured, plated, and transfected as previously described (Glantz et al., 2018). Cells were imaged 24 h after transfection.

#### Microscopy calibration and assays

##### Widefield microscopy and spatial patterned excitation

Widefield imaging was performed on an automated epifluorescence microscope equipped with a custom-built spatial patterning system (Berlew et al., 2020) to deliver blue light (λ = 455/20 nm, 12 mW/cm^2^, 100-1000 ms pulses at duty cycle = 0.8–10%). Excitation patterns were typically 25 μm-wide squares that illuminated ∼25-50% of selected cell area.

##### Spinning disk confocal microscopy

A Nikon Eclipse Ti2 microscope equipped with a CSU-W1 confocal scanner unit (Yokogawa) was used with a 60x/1.4 NA objective. BcLOV4 was stimulated at λ = 405 nm at either 50% [12.24 W/cm^2^ at the focal plane] or 100% [16.94 W/cm^2^ at the focal plane] laser power. mCherry was excited at λ = 552 nm and imaged using a Brightline mCherry-C-000 filter cube (562/40 excitation filter, 593 dichroic, and 641/75 emission filter). When necessary, spatially patterned excitation could be delivered via a Nikon DMD module (tunable irradiance between 10 and 70 mW/cm^2^ at the focal plane). In general, excitation intensity was set at ∼12 mW/cm^2^ to allow for accurate inter-comparison between widefield and confocal DMD excitation datasets.

##### Excitation intensity measurements

For widefield wholefield, widefield DMD excitation, and confocal DMD excitation, intensity at the focal plane was measured with a Thorlabs PM100D power meter. PyOptica (Grochowicz and Miler, 2020) was used to calculate the expected out-of-plane excitation or light intensity at different z-locations that would yield the observed DMD image formation at the focal plane. Because of the underfilling of the back-aperture of the objective, the excitation volume for DMD excitation is, at μm-distance scales, a vertical square column of light with identical irradiance profiles in each z-plane. This approximation was used for all DMD simulations.

For wholefield spinning disk confocal excitation, illumination intensity at the focal plane was measured by fitting photobleaching curves of <5 μm water-in-oil emulsion droplets filled with 100 μM riboflavin. To estimate out-of-plane excitation, we approximated the spinning disk unit as continuously scanning multiple diffraction-limited Gaussian beams across the field-of-view (neglecting any beam-shaping occurring in the unit). The intensity of a Gaussian beam can be modeled as (Dickson, 1970):

(Equation 9) I(r,z)=I0(w0w(z)2)e−2r2w(z)2

where $r=\sqrt{x^{2}+y^{2}}$ and w is the beam radius and is defined as:

(Equation 10) w(z)=w01+(zzR)2

where $z_{R}$ is the Rayleigh range:

(Equation 11) zR=πw02nsampleλ

where *n*_*sample*_ is the refractive index of the propagating medium and λ is the wavelength of the light. Note that *w*_*0*_ can be estimated from the point spread function (calculated as 109.8 nm). The time-averaged volumetric intensity distribution of delivered excitation light was then approximated as the convolution of *I*(*r*,*z*) with a 2D kernel representing the amount of time the spinning disk scanner spends at each point. For whole-field excitation, the average light intensity that out-of-focus planes of the cell are exposed to was found to be approximately equal to the light intensity at the focal plane and hence a uniform excitation light intensity throughout the cell volume was assumed in corresponding models.

##### Point spread function measurements

Spinning disk confocal PSFs were measured by taking z-stacks of 100 nm fluorescent beads (Degradex 2101C/2211). Widefield PSFs were measured using larger beads 100-390 nm fluorescent beads (Spherotech FP-0252-2/FCM-02556-2). PSFs were extracted from z-stacks using the Python Microscopy Environment (Baddeley et al., 2021) and subsequently de-convolved to account for non-negligible bead diameter. 3D Gaussian (spinning disk) or Richards & Wolf (widefield) PSF models were fit to the experimentally derived PSFs with the python PSF package (Gohlke and Holub, 2021) to limit noise for subsequent analysis.

##### In cellulo measurement of kinetic constants

The values for *k*_*on*,*lit*_, *k*_*off*,*lit*_, *k*_*on*,*dark*_, and *k*_*off*, *dark*_, were extracted from *in situ* HEK cell measurements. Given the fast cytosolic diffusion relative to the membrane binding rate of BcLOV4, binding was modeled as reaction-limited, and assuming isotropic diffusion, was simplified to a four-state zero-dimensional model:

(Equation 12) duCdt=kon,pvC−koff,puC+koff,litLuM−kon,litL(Smax−uM−vM)uCdvCdt=−kon,pvC+koff,puC+koff,darkLvM−kon,darkL(Smax−uM−vM)vCduMdt=kon,pvM−koff,puM−koff,lituM+kon,lit(Smax−uM−vM)uCdvMdt=koff,puM−kon,pvM−koff,darkvM+kon,dark(Smax−uM−vM)vC

where *L* is a characteristic length-constant, defined as the volume-to-surface area ratio of the cell, which is necessary to maintain conservation of mass in the system. *L* is directly measurable via confocal imaging.

For BcLOV4 membrane unbinding post-excitation when t≫1koff,p, $u_{M},u_{C}\approx 0$ and the model can be simplified to:

(Equation 13) dvCdt=−koff,darkLvM−kon,darkL(Smax−vM)vCdvMdt=−koff,darkvM+kon,dark(Smax−vM)vC

Due to the relatively small expected value of *k*_*on*,*dark*_, it is neglected to recover the expression:

(Equation 14) $$v_{M}=v_{M,0}e^{-k_{off,dark}t}$$

Based on simulations, the model was accurate to within 5% for t> 40 s post-completion of excitation. *k*_*off*, *dark*_ was thus determined from a spinning-disk dataset of cytosolic fluorescence recovery after 5 s wholefield excitation of BcLOV4 at 16.94 W/cm^2^ (Figures S4E and S4F). To fit *k*_*on*,*lit*_ and *k*_*off*,*lit*_, the 0D-model in Equation (12) was fit to a spinning disk dataset of dark-adapted BcLOV4 stimulated 100 ms every 1 s (10% duty cycle) at 16.94 W/cm^2^ (Figures S4A–S4D).

Due to relative model insensitivity to the value of *k*_*on*,*dark*_, which primarily affects dark-state affinity and has minimal effect on membrane binding or unbinding kinetics, it was better estimated as a bound. Given the fact that BcLOV4 at the membrane could not be visualized in the dark state regardless of cytosolic BcLOV4 concentration, it followed that membrane fluorescence (*F*_*mem*_) was always less than or equal to cytosolic fluorescence (*F*_*cyto*_), i.e., *F*_*mem*_ ≤ *F*_*cyto*_. *F*_*mem*_ is approximately a linear function of the protein density at the membrane, which in turn is related to the dark-state BcLOV4 concentration by the Langmuir isotherm. Hence:

(Equation 15) $$F_{mem}=A_{1}\frac{S_{max}{\left[BcLOV4\right]}_{dark}}{{\left[BcLOV4\right]}_{dark}+K_{D,dark}}+A_{2}$$

$A_{1}$, $\;A_{2}$ were determined using the giant unilamellar vesicles (GUV) calibration (see “Surface density calibration”). Similarly, for *F*_*cyto*_:

(Equation 16) $$F_{cyto}=B_{1}{\left[BcLOV4\right]}_{dark}+B_{2}$$

where $B_{1}$, $\;B_{2}$ were measured with patch micropipette microinjection of Lucifer Yellow (see “Concentration calibration”). Assuming *F*_*mem*_ ≤ *F*_*cyto*_, the lower bound on *K*_*D*,*dark*_ of 20 μM was determined from Equations 15 and 16, and consequently the upper bound on *k*_*on*,*dark*_ of 1125 M^−1^s^−1^, was used in the model as a “worst-case” value (Figure S4G).

##### Surface density calibration

The conversion between BcLOV4 surface density and membrane fluorescence was measured based on techniques by others (Dezi et al., 2013). Briefly, phosphatidylcholine giant unilamellar vesicles (GUVs) with varying concentrations of Texas Red-1,2-dihexadecanoyl-*sn*-glycero-3-phosphoethanolamine, triethylammonium salt (Tx-DHPE, Biotium) between 0.05 and 1% mole/mole were generated using the water-in-oil emulsion transfer method (Nishimura et al., 2012). GUVs were imaged at their equatorial plane by spinning-disk confocal microscopy, and a calibration curve was constructed which related Tx-DHPE density to measured membrane fluorescence. GUVs were automatically identified in the image by the circle Hough transform and membrane fluorescence was extracted in MATLAB as the average signal measured along the circumference. The relative brightness of Texas Red versus mCherry under our imaging conditions converted this to a curve of BcLOV4 density versus membrane fluorescence. On the spinning disk confocal microscope used here, the measured membrane fluorescence is 1.2-fold the BcLOV4 surface density in molecules/μm^2^.

##### Concentration calibration

Due to model nonlinearities, all inputs and outputs need to be expressed in absolute concentration units. The fluorescence-derived concentrations were measured based on previously used methods (Cherkas et al., 2018), wherein a known concentration of dye is delivered to cells by whole-cell patch-clamp (Figure S1A). HEK cells’ transmembrane potentials were kept stable using an Axopatch 200B amplifier and Digidata 1440 digitizer (Molecular Devices) at room temperature. Patch micropipettes were pulled to obtain a resistance of 3-10 MΩ (1.5 × 0.86 mm borosilicate glass, Sutter Instrument P-1000 pipette puller). Extracellular solution (Tyrode’s solution) consisted of 125 mM NaCl, 2 mM KCl, 3 mM CaCl_2_, 1 mM MgCl_2_, 10 mM HEPES, 30 mM glucose, pH 7.3 (NaOH adjusted), 300 mOsm (sucrose adjusted). Intracellular solution consisted of 125 mM K-gluconate, 8 mM NaCl, 0.1 mM CaCl_2_, 0.6 mM MgCl_2_, 1 mM EGTA, 10 mM HEPES, 4 mM Mg-ATP, 0.4 mM Na-GTP, pH 7.3 (KOH adjusted), with 295–300 mOsm (sucrose adjusted). Intracellular solution was supplemented with Lucifer Yellow (LY) dye (5-25 μM) for perfusion. Cells with a leak current > 200 pA or access resistance >25 MΩ were discarded.

Time-lapse fluorescent imaging was conducted at 6 Hz to monitor LY filling of the cell cytoplasm, which took ∼5 min to saturate. (N = 3 cells per 5-25 μM LY concentration). Known concentrations of BcLOV4-mCherry and LY were imaged in bulk solution, to derive the relative fluorescence ratio between these two fluorophores for the microscope. With this information, a linear function was constructed to approximate cellular BcLOV4 concentration (in μM) from fluorescence visualization intensity, which was given by F=505[BcLOV4]+125 for the widefield microscope and F=452[BcLOV4]+107 for the spinning-disk confocal microscope, given the imaging parameters used herein.

Based on these empirical relationships, the distribution of cellular BcLOV4 concentrations after transient transfection were determined from confocal data (Figure S1B) to be a right-ward skewed distribution with a mean concentration of 4.02 μM. As secondary validation of this measurement, mCherry fluorescence in HEK cell lysate (SoluLyse-M, Amsbio) was determined from calibrated fluorescence of FPLC-purified recombinant BcLOV4-mCherry, after correcting for cell confluency, transfection efficiency, and cell volume/lysate volume; the confirmatory calculated mean concentration was 3.72 ± 0.79 μM (N = 4).

##### FRAP measurements

FRAP was performed using a Leica TCS SP8 laser scanning confocal microscope (Figure S3). For lateral membrane diffusion, BcLOV4 was initially recruited to the membrane with a 100 ms pulse of 405 nm light. A small rectangular ROI (average size ∼1.5 × 0.5 μm, with the long axis parallel to the membrane) on the membrane was photobleached using a high intensity 561 nm laser at 100% power. The ROI was then imaged at ∼60 Hz until its average fluorescence stabilized. When fit to a 1D reaction-diffusion model that accounted for membrane unbinding/rebinding, the membrane unbinding/rebinding could account for most of the observed fluorescence recovery, resulting in *D*_*mem*_ values below the detection limit. Therefore, the effective upper bound on *D*_*mem*_ was determined by fitting the data to a canonical model without the reaction components (Ellenberg et al., 1997):

(Equation 17) $$I\left(t\right)=I_{final}{\left(1-w^{2}{\left(w^{2}+4\pi D_{mem}t\right)}^{-1}\right)}^{1/2}$$

where *w* is the width of the bleaching rectangle, and *I* is the intensity within the ROI.

For measurement of cytosolic diffusion, a 2 μm diameter circular ROI near the center of the cell was photobleached using a high intensity 561 nm laser at 100% power (∼250 ms bleach time). Fluorescence recovery was measured in the Leica “fly mode” to capture the initial few ms of the recovery curve and tracked at ∼600 Hz until the signal stabilized. The half-time of recovery was extracted from the data through fitting of an exponential recovery curve, to calculate *D*_*cyto*_ using a previously reported conversion for circular ROIs (Kang et al., 2012).

##### Spatial confinement

Spatial confinement experiments were conducted using a Leica TCS SP8 laser scanning confocal microscope. A dark-adapted cell was imaged at ∼5 Hz for 4 s, after which a small perimembrane ROI (∼1.5 x ∼0.5 μm) was photoexcited with a ∼200 ms pulse of 405 nm laser light. The cell was subsequently imaged at ∼5 Hz until the fluorescence in the ROI returned to baseline. The ROI and membrane profile fluorescence kinetics were extracted using a custom-written ImageJ macro. Results were compared to model predictions which utilized the experimental cell geometry. Gaussian curve fitting was conducted in MATLAB.

#### Simulations

##### Mesh initialization and quality control

For all simulations, unless otherwise specified, all cytosolic mesh nodes were initialized with the cellular cytosolic concentration calculated from the input fluorescence micrograph. Concentration was assumed to be uniform in the cytoplasm because z-stacks analysis did not show variability that was not primarily attributable to organelle voids, cell geometry, or diffraction. Membrane mesh nodes were initialized as $S_{max}K_{D,dark}/\left(K_{D,dark}+{\left[BcLOV4\right]}_{cyto}\right)$. The volume of mesh elements was <5 μm^3^. Mesh size was selected so that, on average, a <1% error relative to super-fine mesh (∼10^−4^ μm^3^) was maintained (Figures S2A and S2B). Timesteps varied from 0.01-0.1 s and were selected so that a <1% error relative to a timestep of 0.001 s was achieved (Figure S2C). As secondary validation, in all cases, it was verified that the resultant solution did not change with a finer mesh or smaller timestep.

##### Spatial discretization using FEM

The approach is based on bulk-surface reaction-diffusion systems (Madzvamuse and Chung, 2016; Cusseddu et al., 2019; Paquin-Lefebvre et al., 2020). As shorthand, $S=S_{max}-u_{M}-v_{M}.\;$ The weak form of this system defined by Equations (1) and (2) is (letting φ,ψ be our test functions on Ω and dΩ, respectively):

(Equation 18) $$\begin{array}{l} \underset{\text{Ω}}{\int }\frac{du_{C}}{dt}\varphi +D_{cyto}\underset{\text{Ω}}{\int }\nabla u_{C}\cdot \nabla \varphi =\\ \underset{\text{Ω}}{\int }\left(k_{on,p}v_{C}-k_{off,p}u_{C}\right)\varphi +\underset{d\text{Ω}}{\int }\left(k_{off,lit}u_{M}-k_{on,lit}Su_{C}\right)\varphi \\ \underset{\text{Ω}}{\int }\frac{dv_{C}}{dt}\varphi +D_{cyto}\underset{\text{Ω}}{\int }\nabla v_{C}\cdot \nabla \varphi =\\ \underset{\text{Ω}}{\int }\left(-k_{on,p}v_{C}+k_{off,p}u_{C}\right)\varphi +\underset{d\text{Ω}}{\int }\left(k_{off,dark}v_{M}-k_{on,dark}Sv_{C}\right)\varphi \\ \underset{d\text{Ω}}{\int }\frac{du_{M}}{dt}\psi +D_{mem}\underset{d\text{Ω}}{\int }\nabla_{d\text{Ω}}u_{M}\cdot \nabla_{d\text{Ω}}\psi =\\ \underset{d\text{Ω}}{\int }\left(k_{on,p}v_{M}-\left(k_{off,p}+k_{off,lit}\right)u_{M}+k_{on,lit}Su_{C}\right)\psi \\ \underset{d\text{Ω}}{\int }\frac{du_{C}}{dt}\psi +D_{mem}\underset{d\text{Ω}}{\int }\nabla_{d\text{Ω}}v_{M}\cdot \nabla_{d\text{Ω}}\psi =\\ \underset{d\text{Ω}}{\int }\left(k_{off,p}u_{M}-\left(k_{on,p}+k_{off,dark}\right)v_{M}+k_{on,dark}Sv_{C}\right)\psi \end{array}$$

where $\nabla_{d\text{Ω}}$ is the gradient on dΩ. To spatially discretize the weak form. we define the discretization’s as ${\text{Ω}}_{h}\subset \text{Ω}$ and $d{\text{Ω}}_{h}\subset d\text{Ω}$

(Equation 19) $$\begin{array}{l} \int_{{\text{Ω}}_{h}}\frac{du_{h,C}}{dt}\varphi_{h}+D_{cyto}\int_{{\text{Ω}}_{h}}\nabla u_{h,C}\cdot \nabla \varphi_{h}=\\ \int_{{\text{Ω}}_{h}}\left(k_{on,p}v_{h,C}-k_{off,p}u_{h,C}\right)\varphi_{h}+\int_{{\text{Ω}}_{h}}\left(k_{off,lit}u_{h,M}-k_{on,lit}Su_{h,C}\right)\varphi_{h}\\ \int_{{\text{Ω}}_{h}}\frac{dv_{h,C}}{dt}\varphi_{h}+D_{cyto}\int_{{\text{Ω}}_{h}}\nabla v_{h,C}\cdot \nabla \varphi_{h}=\\ \underset{{\text{Ω}}_{h}}{\int }\left(-k_{on,p}v_{h,C}+k_{off,p}u_{h,C}\right)\varphi_{h}+\underset{d{\text{Ω}}_{h}}{\int }\left(k_{off,dark}v_{h,M}-k_{on,dark}Sv_{h,C}\right)\varphi_{h}\\ \underset{d{\text{Ω}}_{h}}{\int }\frac{du_{h,M}}{dt}\psi_{h}+D_{mem}\underset{d{\text{Ω}}_{h}}{\int }\nabla_{d\text{Ω}}u_{h,M}\cdot \nabla_{d\text{Ω}}\psi_{h}=\\ \underset{d{\text{Ω}}_{h}}{\int }\left(k_{on,p}v_{h,M}-\left(k_{off,p}+k_{off,l}\right)u_{h,M}+k_{on,l}Su_{h,C}\right)\psi_{h}\\ \underset{d{\text{Ω}}_{h}}{\int }\frac{dv_{h,M}}{dt}\psi_{h}+D_{mem}\underset{d{\text{Ω}}_{h}}{\int }\nabla_{d\text{Ω}}v_{h,M}\cdot \nabla_{d\text{Ω}}\psi_{h}=\\ \underset{d{\text{Ω}}_{h}}{\int }\left(k_{off,p}u_{h,M}-\left(k_{on,p}+k_{off,dark}\right)v_{h,M}+k_{on,dark}Sv_{h,C}\right)\psi_{h} \end{array}$$

In matrix form, we can express this as:

(Equation 20) $$\begin{array}{l} K_{\varphi }\frac{d{\hat{u}}_{C}}{dt}+D_{cyto}A_{\varphi }{\hat{u}}_{C}-k_{on,p}K_{\varphi }{\hat{v}}_{C}+k_{off,p}K_{\varphi }{\hat{u}}_{C}-k_{off,lit}K_{\psi \varphi }{\hat{u}}_{M}+k_{on,lit}\left(S_{max}K_{\varphi \varphi }-B_{\varphi }\left({\hat{u}}_{M}\right)-B_{\varphi }\left({\hat{v}}_{M}\right)\right){\hat{u}}_{C}=0\\ K_{\varphi }\frac{d{\hat{v}}_{C}}{dt}+D_{cyto}A_{\varphi }{\hat{v}}_{C}+k_{on,p}K_{\varphi }{\hat{v}}_{C}-k_{off,p}K_{\varphi }{\hat{u}}_{C}-k_{off,dark}K_{\psi \varphi }{\hat{v}}_{M}+k_{on,dark}\left(S_{max}K_{\varphi \varphi }-B_{\varphi }\left({\hat{u}}_{M}\right)-B_{\varphi }\left({\hat{v}}_{M}\right)\right){\hat{v}}_{C}=0\\ K_{\psi }\frac{d{\hat{u}}_{M}}{dt}+D_{mem}A_{\psi }{\hat{u}}_{M}-k_{on,p}K_{\psi }{\hat{v}}_{M}+\left(k_{off,p}+k_{off,lit}\right)K_{\psi }{\hat{u}}_{M}-k_{on,lit}\left(S_{max}K_{\varphi \psi }-B_{\psi }\left({\hat{u}}_{M}\right)-B_{\psi }\left({\hat{v}}_{M}\right)\right){\hat{u}}_{C}=0\\ K_{\psi }\frac{d{\hat{v}}_{M}}{dt}+D_{mem}A_{\psi }{\hat{v}}_{M}-k_{off,p}K_{\psi }{\hat{u}}_{M}+\left(k_{on,p}+k_{off,dark}\right)K_{\psi }{\hat{v}}_{M}-k_{on,dark}\left(S_{max}K_{\varphi \psi }-B_{\psi }\left({\hat{u}}_{M}\right)-B_{\psi }\left({\hat{v}}_{M}\right)\right){\hat{v}}_{C}=0 \end{array}$$

where:

(Equation 21) $$\begin{array}{l} {\left(K_{\varphi }\right)}_{ij}=\int_{\Omega }\varphi_{i}\varphi_{j}\\ {\left(K_{\psi }\right)}_{ij}=\underset{d\text{Ω}}{\int }\psi_{i}\psi_{j}\\ {\left(K_{\varphi \varphi }\right)}_{ij}=\underset{d\text{Ω}}{\int }\varphi_{i}\varphi_{j}\\ {\left(K_{\psi \varphi }\right)}_{ij}=\underset{d\text{Ω}}{\int }\psi_{i}\varphi_{j}\\ {\left(K_{\varphi \psi }\right)}_{ij}=\underset{d\text{Ω}}{\int }\varphi_{i}\psi_{j}\\ {\left(A_{\varphi }\right)}_{ij}=\underset{\text{Ω}}{\int }\nabla \varphi_{i}\cdot \nabla \varphi_{j}\\ {\left(A_{\psi }\right)}_{ij}=\underset{d\text{Ω}}{\int }\nabla_{d\text{Ω}}\psi_{i}\cdot \nabla_{d\text{Ω}}\psi_{j}\\ {\left(B_{\varphi }\left(x\right)\right)}_{ij}=\underset{d\text{Ω}}{\int }\left(x\cdot \psi \right)\varphi_{i}\varphi_{j}\\ {\left(B_{\psi }\left(x\right)\right)}_{ij}=\underset{d\text{Ω}}{\int }\left(x\cdot \psi \right)\varphi_{i}\psi_{j} \end{array}$$

##### Time discretization and integration

For time integration, the fractional theta method was used, which is an implicit method with the A-stability of implicit Euler and second order temporal convergence of Crank-Nicholson (Chrispell et al., 2007; Madzvamuse and Chung, 2014). In this method, the desired time interval is first split into equal length steps, henceforth termed τ. For each time step, 3 sub-steps are performed. The equations to be solved for each time step are outlined below, where θ=1−12.

The first sub-step solves the following linear equations:

(Equation 22) $$\begin{array}{l} K_{\varphi }\frac{\left({\hat{u}}_{C}^{n+\theta }-{\hat{u}}_{C}^{n}\right)}{\theta \tau }+D_{cyto}A_{\varphi }{\hat{u}}_{C}^{n+\theta }-k_{on,p}K_{\varphi }{\hat{v}}_{C}^{n+\theta }+k_{off,p}K_{\varphi }{\hat{u}}_{C}^{n+\theta }-k_{off,lit}K_{\psi \varphi }{\hat{u}}_{M}^{n+\theta }+k_{on,lit}S_{max}K_{\varphi \varphi }{\hat{u}}_{C}^{n+\theta }=k_{on,lit}\left(B_{\varphi }\left({\hat{u}}_{M}^{n}\right)+B_{\varphi }\left({\hat{v}}_{M}^{n}\right)\right){\hat{u}}_{C}^{n}\\ K_{\varphi }\frac{\left({\hat{v}}_{C}^{n+\theta }-{\hat{v}}_{C}^{n}\right)}{\theta \tau }+D_{cyto}A_{\varphi }{\hat{v}}_{C}^{n+\theta }+k_{on,p}K_{\varphi }{\hat{v}}_{C}^{n+\theta }-k_{off,p}K_{\varphi }{\hat{u}}_{C}^{n+\theta }-k_{off,dark}K_{\psi \varphi }{\hat{v}}_{M}^{n+\theta }+k_{on,dark}S_{max}K_{\varphi \varphi }{\hat{v}}_{C}^{n+\theta }=k_{on,dark}(B_{\varphi }\left({\hat{u}}_{M}^{n+\theta }\right)+\\ B_{\varphi }\left({\hat{v}}_{M}^{n+\theta }\right)){\hat{v}}_{C}^{n+\theta }\\ K_{\psi }\frac{\left({\hat{u}}_{M}^{n+\theta }-{\hat{u}}_{M}^{n}\right)}{\theta \tau }+D_{mem}A_{\psi }{\hat{u}}_{M}^{n+\theta }-k_{on,p}K_{\psi }{\hat{v}}_{M}^{n+\theta }+\left(k_{off,p}+k_{off,lit}\right)K_{\psi }{\hat{u}}_{M}^{n+\theta }-k_{on,lit}S_{max}K_{\varphi \psi }{\hat{u}}_{C}^{n+\theta }=-k_{on,lit}\left(B_{\psi }\left({\hat{u}}_{M}^{n+\theta }\right)+B_{\psi }\left({\hat{v}}_{M}^{n+\theta }\right)\right){\hat{u}}_{C}^{n+\theta }\\ K_{\psi }\frac{\left({\hat{v}}_{M}^{n+\theta }-{\hat{v}}_{M}^{n}\right)}{\theta \tau }+D_{mem}A_{\psi }{\hat{v}}_{M}^{n+\theta }-k_{off,p}K_{\psi }{\hat{u}}_{M}^{n+\theta }+\left(k_{on,p}+k_{off,dark}\right)K_{\psi }{\hat{v}}_{M}^{n+\theta }-k_{on,dark}S_{max}K_{\varphi \psi }{\hat{v}}_{C}^{n+\theta }=-k_{on,dark}\left(B_{\psi }\left({\hat{u}}_{M}^{n+\theta }\right)+B_{\psi }\left({\hat{v}}_{M}^{n+\theta }\right)\right){\hat{v}}_{C}^{n+\theta } \end{array}$$

The second sub-step solves the following non-linear equations:

(Equation 23) $$\begin{array}{l} K_{\varphi }\frac{\left({\hat{u}}_{C}^{n+1-\theta }-{\hat{u}}_{C}^{n+\theta }\right)}{\left(1-2\theta \right)\tau }-k_{on,lit}\left(B_{\varphi }\left({\hat{u}}_{M}^{n+1-\theta }\right)+B_{\varphi }\left({\hat{v}}_{M}^{n+1-\theta }\right)\right){\hat{u}}_{C}^{n+1-\theta }=-D_{cyto}A_{\varphi }{\hat{u}}_{C}^{n+\theta }+k_{on,p}K_{\varphi }{\hat{v}}_{C}^{n+\theta }-k_{off,p}K_{\varphi }{\hat{u}}_{C}^{n+\theta }+k_{off,lit}K_{\psi \varphi }{\hat{u}}_{M}^{n+\theta }-k_{on,lit}S_{max}K_{\varphi \varphi }{\hat{u}}_{C}^{n+\theta }\\ K_{\varphi }\frac{\left({\hat{v}}_{C}^{n+1-\theta }-{\hat{v}}_{C}^{n+\theta }\right)}{\left(1-2\theta \right)\tau }-k_{on,dark}\left(B_{\varphi }\left({\hat{u}}_{M}^{n+1-\theta }\right)+B_{\varphi }\left({\hat{v}}_{M}^{n+1-\theta }\right)\right){\hat{v}}_{C}^{n+1-\theta }=-D_{cyto}A_{\varphi }{\hat{v}}_{C}^{n+\theta }-k_{on,p}K_{\varphi }{\hat{v}}_{C}^{n+\theta }+k_{off,p}K_{\varphi }{\hat{u}}_{C}^{n+\theta }+k_{off,dark}K_{\psi \varphi }{\hat{v}}_{M}^{n+\theta }-k_{on,dark}S_{max}K_{\varphi \varphi }{\hat{v}}_{C}^{n+\theta }\\ K_{\psi }\frac{\left({\hat{u}}_{M}^{n+1-\theta }-{\hat{u}}_{M}^{n+\theta }\right)}{\left(1-2\theta \right)\tau }+k_{on,lit}\left(B_{\psi }\left({\hat{u}}_{M}^{n+1-\theta }\right)+B_{\psi }\left({\hat{v}}_{M}^{n+1-\theta }\right)\right){\hat{u}}_{C}^{n+1-\theta }=-D_{mem}A_{\psi }{\hat{u}}_{M}^{n+\theta }+k_{on,p}K_{\psi }{\hat{v}}_{M}^{n+\theta }-\left(k_{off,p}+k_{off,lit}\right)K_{\psi }{\hat{u}}_{M}^{n+\theta }+k_{on,lit}S_{max}K_{\varphi \psi }{\hat{u}}_{C}^{n+\theta }\\ K_{\psi }\frac{\left({\hat{v}}_{M}^{n+1-\theta }-{\hat{v}}_{M}^{n+\theta }\right)}{\left(1-2\theta \right)\tau }+k_{on,dark}\left(B_{\psi }\left({\hat{u}}_{M}^{n+1-\theta }\right)-B_{\psi }\left({\hat{v}}_{M}^{n+1-\theta }\right)\right){\hat{v}}_{C}^{n+1-\theta }=-D_{mem}A_{\psi }{\hat{v}}_{M}^{n+\theta }+k_{off,p}K_{\psi }{\hat{u}}_{M}^{n+\theta }-\left(k_{on,p}+k_{off,dark}\right)K_{\psi }{\hat{v}}_{M}^{n+\theta }+k_{on,dark}S_{max}K_{\varphi \psi }{\hat{v}}_{C}^{n+\theta } \end{array}$$

The third sub-step solves the following linear system:

(Equation 24) $$\begin{array}{l} K_{\varphi }\frac{\left({\hat{u}}_{C}^{n+1}-{\hat{u}}_{C}^{n+1-\theta }\right)}{\theta \tau }+D_{cyto}A_{\varphi }{\hat{u}}_{C}^{n+1}-k_{on,p}K_{\varphi }{\hat{v}}_{C}^{n+1}+k_{off,p}K_{\varphi }{\hat{u}}_{C}^{n+1}-k_{off,lit}K_{\psi \varphi }{\hat{u}}_{M}^{n+1}+k_{on,l}itS_{max}K_{\varphi \varphi }{\hat{u}}_{C}^{n+1}=-k_{on,lit}\left(B_{\varphi }\left({\hat{u}}_{M}^{n+1-\theta }\right)+B_{\varphi }\left({\hat{v}}_{M}^{n+1-\theta }\right)\right){\hat{u}}_{C}^{n+1-\theta }\\ K_{\varphi }\frac{\left({\hat{v}}_{C}^{n+1}-{\hat{v}}_{C}^{n+1-\theta }\right)}{\theta \tau }+D_{cyto}A_{\varphi }{\hat{v}}_{C}^{n+1}+k_{on,p}K_{\varphi }{\hat{v}}_{C}^{n+1}-k_{off,p}K_{\varphi }{\hat{u}}_{C}^{n+1}-k_{off,dark}K_{\psi \varphi }{\hat{v}}_{M}^{n+1}+k_{on,dark}S_{max}K_{\varphi \varphi }{\hat{v}}_{C}^{n+1}=k_{on,dark}(B_{\varphi }\left({\hat{u}}_{M}^{n+1-\theta }\right)+B_{\varphi }\left({\hat{v}}_{M}^{n+1-\theta }\right)){\hat{v}}_{C}^{n+1-\theta }\\ K_{\psi }\frac{\left({\hat{u}}_{M}^{n+1}-{\hat{u}}_{M}^{n+1-\theta }\right)}{\theta \tau }+D_{mem}A_{\psi }{\hat{u}}_{M}^{n+\theta }-k_{on,p}K_{\psi }{\hat{v}}_{M}^{n+\theta }+\left(k_{off,p}+k_{off,lit}\right)K_{\psi }{\hat{u}}_{M}^{n+\theta }-k_{on,lit}S_{max}K_{\varphi \psi }{\hat{u}}_{C}^{n+\theta }=-k_{on,lit}\left(B_{\psi }\left({\hat{u}}_{M}^{n+1-\theta }\right)+B_{\psi }\left({\hat{v}}_{M}^{n+1-\theta }\right)\right){\hat{u}}_{C}^{n+1-\theta }\\ K_{\psi }\frac{\left({\hat{v}}_{M}^{n+1}-{\hat{v}}_{M}^{n+1-\theta }\right)}{\theta \tau }+D_{mem}A_{\psi }{\hat{v}}_{M}^{n+1}-k_{off,p}K_{\psi }{\hat{u}}_{M}^{n+1}+\left(k_{on,p}+k_{off,dark}\right)K_{\psi }{\hat{v}}_{M}^{n+1}-k_{on,dark}S_{max}K_{\varphi \psi }{\hat{v}}_{C}^{n+1}=-k_{on,dark}\left(B_{\psi }\left({\hat{u}}_{M}^{n+1-\theta }\right)-B_{\psi }\left({\hat{v}}_{M}^{n+1-\theta }\right)\right){\hat{v}}_{C}^{n+1-\theta } \end{array}$$

Although sub-steps 1 and 3 involve easily solvable linear systems, sub-step 2 involves a non-linear system. We solve this system using a Newton-Raphson iteration. For this problem, the Jacobian is defined as follows below. Given the following residuals:

(Equation 25) $$\begin{array}{l} K_{\varphi }\frac{\left({\hat{u}}_{C}^{n+1-\theta }-{\hat{u}}_{C}^{n+\theta }\right)}{\left(1-2\theta \right)\tau }-k_{on,lit}\left(B_{\varphi }\left({\hat{u}}_{M}^{n+1-\theta }\right)+B_{\varphi }\left({\hat{v}}_{M}^{n+1-\theta }\right)\right){\hat{u}}_{C}^{n+1-\theta }+D_{cyto}A_{\varphi }{\hat{u}}_{C}^{n+\theta }-k_{on,p}K_{\varphi }{\hat{v}}_{C}^{n+\theta }+k_{off,p}K_{\varphi }{\hat{u}}_{C}^{n+\theta }-k_{off,lit}K_{\psi \varphi }{\hat{u}}_{M}^{n+\theta }+k_{on,lit}S_{max}K_{\varphi \varphi }{\hat{u}}_{C}^{n+\theta }=0\\ K_{\varphi }\frac{\left({\hat{v}}_{C}^{n+1-\theta }-{\hat{v}}_{C}^{n+\theta }\right)}{\left(1-2\theta \right)\tau }-k_{on,dark}\left(B_{\varphi }\left({\hat{u}}_{M}^{n+1-\theta }\right)+B_{\varphi }\left({\hat{v}}_{M}^{n+1-\theta }\right)\right){\hat{v}}_{C}^{n+1-\theta }+D_{cyto}A_{\varphi }{\hat{v}}_{C}^{n+\theta }+k_{on,p}K_{\varphi }{\hat{v}}_{C}^{n+\theta }-k_{off,p}K_{\varphi }{\hat{u}}_{C}^{n+\theta }-k_{off,dark}K_{\psi \varphi }{\hat{v}}_{M}^{n+\theta }+k_{on,dark}S_{max}K_{\varphi \varphi }{\hat{v}}_{C}^{n+\theta }=0\\ K_{\psi }\frac{\left({\hat{u}}_{M}^{n+1-\theta }-{\hat{u}}_{M}^{n+\theta }\right)}{\left(1-2\theta \right)\tau }+k_{on,lit}\left(B_{\psi }\left({\hat{u}}_{M}^{n+1-\theta }\right)+B_{\psi }\left({\hat{v}}_{M}^{n+1-\theta }\right)\right){\hat{u}}_{C}^{n+1-\theta }+D_{mem}A_{\psi }{\hat{u}}_{M}^{n+\theta }-k_{on,p}K_{\psi }{\hat{v}}_{M}^{n+\theta }+\left(k_{off,p}+k_{off,lit}\right)K_{\psi }{\hat{u}}_{M}^{n+\theta }-k_{on,lit}S_{max}K_{\varphi \psi }{\hat{u}}_{C}^{n+\theta }=0\\ K_{\psi }\frac{\left({\hat{v}}_{M}^{n+1-\theta }-{\hat{v}}_{M}^{n+\theta }\right)}{\left(1-2\theta \right)\tau }+k_{on,dark}\left(B_{\psi }\left({\hat{u}}_{M}^{n+1-\theta }\right)-B_{\psi }\left({\hat{v}}_{M}^{n+1-\theta }\right)\right){\hat{v}}_{C}^{n+1-\theta }+D_{mem}A_{\psi }{\hat{v}}_{M}^{n+\theta }-k_{off,p}K_{\psi }{\hat{u}}_{M}^{n+\theta }+\left(k_{on,p}+k_{off,d}\right)K_{\psi }{\hat{v}}_{M}^{n+\theta }-k_{on,dark}S_{max}K_{\varphi \psi }{\hat{v}}_{C}^{n+\theta }=0 \end{array}$$

Constructing the Jacobian as a 4 x 4 block matrix yields the following Jacobian elements:

(Equation 26) $$\begin{array}{l} J_{1,1}=K_{\varphi }-k_{on,lit}\left(1-2\theta \right)\tau \left((B_{\varphi }\left({\hat{u}}_{M}^{n+1-\theta }\right)+B_{\varphi }\left({\hat{v}}_{M}^{n+1-\theta }\right)\right)\\ J_{1,2}=0\\ J_{1,3}=-k_{on,lit}\left(1-2\theta \right)\tau K_{\varphi }{\hat{u}}_{C}^{n+1-\theta }\\ J_{1,4}=-k_{on,lit}\left(1-2\theta \right)\tau K_{\varphi }{\hat{u}}_{C}^{n+1-\theta }\\ J_{2,1}=0\\ J_{2,2}=K_{\varphi }-k_{on,dark}\left(1-2\theta \right)\tau \left((B_{\varphi }\left({\hat{u}}_{M}^{n+1-\theta }\right)+B_{\varphi }\left({\hat{v}}_{M}^{n+1-\theta }\right)\right)\\ J_{2,3}=-k_{on,dark}\left(1-2\theta \right)\tau K_{\varphi }{\hat{v}}_{C}^{n+1-\theta }\\ J_{2,4}=-k_{on,dark}\left(1-2\theta \right)\tau K_{\varphi }{\hat{v}}_{C}^{n+1-\theta }\\ J_{3,1}=k_{on,lit}\left(1-2\theta \right)\tau \left((B_{\psi }\left({\hat{u}}_{M}^{n+1-\theta }\right)+B_{\varphi }\left({\hat{v}}_{M}^{n+1-\theta }\right)\right)\\ J_{3,2}=0\\ J_{3,3}=K_{\psi }+k_{on,lit}\left(1-2\theta \right)\tau K_{\psi }{\hat{u}}_{C}^{n+1-\theta }\\ J_{3,4}=k_{on,lit}\left(1-2\theta \right)\tau K_{\psi }{\hat{u}}_{C}^{n+1-\theta }\\ J_{4,1}=0\\ J_{4,2}=k_{on,dark}\left(1-2\theta \right)\tau \left((B_{\psi }\left({\hat{u}}_{M}^{n+1-\theta }\right)+B_{\psi }\left({\hat{v}}_{M}^{n+1-\theta }\right)\right)\\ J_{4,3}=k_{on,dark}\left(1-2\theta \right)\tau K_{\psi }{\hat{v}}_{C}^{n+1-\theta }a\\ J_{4,4}=K_{\psi }+k_{on,dark}\left(1-2\theta \right)\tau K_{\psi }{\hat{v}}_{C}^{n+1-\theta } \end{array}$$

Using this Jacobian, the equations in sub-step 2 can be solved iteratively.

##### Simplified linear model

A linear version of the model was constructed for purposes of comparison to the full 3D, non-linear model (Figure 3C). For the case of the linear model, $S=S_{max}$. Hence, in matrix form the spatial discretization is (using the previously defined matrix notation):

(Equation 27) $$\begin{array}{l} K_{\varphi }\frac{d{\hat{u}}_{C}}{dt}+D_{cyto}A_{\varphi }{\hat{u}}_{C}-k_{on,p}K_{\varphi }{\hat{v}}_{C}+k_{off,p}K_{\varphi }{\hat{u}}_{C}-k_{off,lit}K_{\psi \varphi }{\hat{u}}_{M}+k_{on,lit}SK_{\varphi \varphi }{\hat{u}}_{C}=0\\ K_{\varphi }\frac{d{\hat{v}}_{C}}{dt}+D_{cyto}A_{\varphi }{\hat{v}}_{C}+k_{on,p}K_{\varphi }{\hat{v}}_{C}-k_{off,p}K_{\varphi }{\hat{u}}_{C}-k_{off,dark}K_{\psi \varphi }{\hat{v}}_{M}+k_{on,dark}SK_{\varphi \varphi }{\hat{v}}_{C}=0\\ K_{\psi }\frac{d{\hat{u}}_{M}}{dt}+D_{mem}A_{\psi }{\hat{u}}_{M}-k_{on,p}K_{\psi }{\hat{v}}_{M}+\left(k_{off,p}+k_{off,lit}\right)K_{\psi }{\hat{u}}_{M}-k_{on,lit}SK_{\varphi \psi }{\hat{u}}_{C}=0\\ K_{\psi }\frac{d{\hat{v}}_{M}}{dt}+D_{mem}A_{\psi }{\hat{v}}_{M}-k_{off,p}K_{\psi }{\hat{u}}_{M}+\left(k_{on,p}+k_{off,dark}\right)K_{\psi }{\hat{v}}_{M}-k_{on,dark}SK_{\varphi \psi }{\hat{v}}_{C}=0 \end{array}$$

To discretize this in time using the backward (implicit) Euler method, we write the following linear system:

(Equation 28) $$\begin{array}{l} K_{\varphi }\frac{\left({\hat{u}}_{C}^{n+1}-{\hat{u}}_{C}^{n}\right)}{\tau }+D_{cyto}A_{\varphi }{\hat{u}}_{C}^{n+1}-k_{on,p}K_{\varphi }{\hat{v}}_{C}^{n+1}+k_{off,p}K_{\varphi }{\hat{u}}_{C}^{n+1}-k_{off,lit}K_{\psi \varphi }{\hat{u}}_{M}^{n+1}+k_{on,lit}SK_{\varphi \varphi }{\hat{u}}_{C}^{n+1}=0\\ K_{\varphi }\frac{\left({\hat{v}}_{C}^{n+1}-{\hat{v}}_{C}^{n}\right)}{\tau }+D_{cyto}A_{\varphi }{\hat{v}}_{C}^{n+1}+k_{on,p}K_{\varphi }{\hat{v}}_{C}^{n+1}-k_{off,p}K_{\varphi }{\hat{u}}_{C}^{n+1}-k_{off,dark}K_{\psi \varphi }{\hat{v}}_{M}^{n+1}+k_{on,dark}SK_{\varphi \varphi }{\hat{v}}_{C}^{n+1}=0\\ K_{\psi }\frac{\left({\hat{u}}_{M}^{n+1}-{\hat{u}}_{M}^{n}\right)}{\tau }+D_{mem}A_{\psi }{\hat{u}}_{M}^{n+1}-k_{on,p}K_{\psi }{\hat{v}}_{M}^{n+1}+\left(k_{off,p}+k_{off,lit}\right)K_{\psi }{\hat{u}}_{M}^{n+1}-k_{on,lit}SK_{\varphi \psi }{\hat{u}}_{C}^{n+1}=0\\ K_{\psi }\frac{\left({\hat{v}}_{M}^{n+1}-{\hat{v}}_{M}^{n}\right)}{\tau }+D_{mem}A_{\psi }{\hat{v}}_{M}^{n+1}-k_{off,p}K_{\psi }{\hat{u}}_{M}^{n+1}+\left(k_{on,p}+k_{off,dark}\right)K_{\psi }{\hat{v}}_{M}^{n+1}-k_{on,dark}SK_{\varphi \psi }{\hat{v}}_{C}^{n+1}=0 \end{array}$$

which can be solved at every timestep to arrive at our desired solution.

##### Cell segmentation

Cell nuclei and plasma membrane contours were detected using the MATLAB active contours algorithm (Chan and Vese, 2001). Briefly, images were initially manually cropped to include only individual cells. Nuclear locations were seeded by drawing a small rectangle in the center of the nuclear void and the nuclear contour was evolved using the rectangle as a starting mask. The plasma membrane contour was evolved by using the entire image as a starting mask. For confocal z stack segmentation, the nuclear/cytoplasmic contours from the previous image in the stack were used as the seeds for the following image. For images where cells were touching (∼80% of widefield images and ∼50% of confocal images), it was necessary to manually crop-out the cells that were not of interest and seed the contours with a hand-drawn contour in ImageJ.

##### Generation of volumetric cell mesh

When taking confocal data 100 nm-step z-stacks of the cell volume were acquired before initiating any experiments to reconstruct a volumetric representation of the cell by interpolating between the segmented 2D frames of the z stack. After the cell volume was constructed, it was converted to a surface triangulation. The surface triangulation was then meshed via MATLAB’s built-in mesh generation algorithm.

In the case of widefield images, which lacked reliable z-stacks because of poor axial resolution, the volumetric representation was constructed by a hemi-ellipsoid projection of the nucleus and cytoplasm from a single 2D frame (Figure 1D). Briefly, the heights for the nucleus and cytoplasm were set as 2 and 3 μm, respectively. The (*x*,*y*) position of the nucleus or cytoplasm ellipsoid zenith and nadir was selected by taking the center-of-mass of a binary mask of the nucleus/cytoplasm. To account for the flat portion of the cell touching the cover glass, the lower portion of the projection was constructed as a hemi-barrel shape. This hemi-barrel was generated by sectioning, with a plane perpendicular to the z axis, a hemi-ellipsoid of height equal to twice the desired height of the hemi-barrel. After the cell volume was constructed, it was converted to a surface triangulation, which was then meshed via MATLAB’s built-in mesh generation algorithm.

##### Sensitivity to biophysical parameters

Upper and lower bounds for *D*_*cyto*_, *D*_*mem*_, *k*_*off*,*p*_, *k*_*on*,*lit*_, *k*_*off*,*lit*_, *k*_*on*,*dark*_, *k*_*off*, *dark*_, and *S*_*max*_ were chosen based on biophysical feasibility and by comparison to values derived for heterodimerization systems. Parameter values were then selected that spanned the space between bounds, yielding 31 total parameter sets where only one parameter at a time differed from BcLOV4’s measured biophysical parameters. Using the same cellular geometry and excitation ROI from Figure 4 and an initial cytosolic protein concentration of 1 μM, the model was then run on each of the 31 parameter sets and analyzed as described in “Spatial confinement.” The parameter *k*_*on*,*p*_ was excluded from the analysis as it does not directly affect spatial confinement.

##### Sensitivity to optical hardware diffraction

To compare the spatial resolution achievable by laser-scanning confocal, two-photon, and TIRF stimulation, a common cell volume was generated by hemi-ellipsoid projection (nuclear height 4 μm, cytoplasmic height 6 μm) of a 2D cell area, where the nucleus was a circle with r = 15 μm and the cytoplasm was a circle with r = 25 μm. Note that the very large cell ensured no artifacts were introduced by protein diffusing all the way around the cell while still accounting for computational memory constraints. Since a very fine mesh was required to ensure sufficient resolution to finely differentiate the excitation volume, the cell volume was meshed to a maximum element size of <0.025 μm^3^ (typically 0.002–0.005 μm^3^), which yielded >10^6^ elements per cell and utilized ∼16 Gb of RAM.

The simulated excitation (λ = 450 nm, 10 W/cm^2^ at the focal plane) region was a 2 μm × 2 μm square centered on the bottom flat portion of the cell volume to allow for reasonably similar conditions for the different hardware. To account for excitation beam rastering within the illumination ROI, one illumination spot for each 100 nm × 100 nm square of the illumination ROI was assumed, for 400 total raster points. The rastering was simulated as being instantaneous since the scan speed is fast relative to the time-course of the experiment.

For each of the microscopy methods, the simulated axial extent and the diffraction-limited nature of the optical beam path were also modeled. TIRF was modeled as using a 63x/1.4 NA objective with an angle of incidence of $\theta_{incidence}$ = 79° and with refractive indices n_sample_ = 1.33, n_coverglass_ = 1.52; then the z-dependence of light intensity was modeled as (Fish, 2009):

(Equation 29) $$I\left(z\right)=I_{0}e^{z/d}$$

where d is given by:

(Equation 30) d=λ4π(ncoverglass2sin2θincidence−nsample2)1/2

where *λ* is the wavelength of excitation light. Equations 9, 10, and 11 were used to model the xy-dependence of the beam, noting that *w*_*0*_, or the waist radius, can be approximated as (Young et al., 2015):

(Equation 31) w0=0.3252(λ(NA0.91+2))

For the case of one-photon stimulation, specifically laser scanning confocal, Equations 9, 10, and 11 were applied to determine the axial dependence, except with the beam waist determined from the PSF as 109.8 nm. For the case of two-photon stimulation, the axial dependence modeling proceeded similarly, except the intensity was given as the square of Equation 9 and λ_2P_ = 2λ_1P_ = 900 nm.

For each of the microscopy methods, we modeled a 100 ms stimulation and simulated 60 s post-stimulation. Unlike for laser-scanning confocal stimulation, the membrane profiles generated via TIRF and two-photon stimulation were not Gaussian; hence, their respective spatial spreads were calculated as the full width at half maximum (FWHM) and then converted to an effective standard deviation via $FWHM=2\sqrt{2ln2}\;STD$, which holds for a Gaussian distribution, to allow comparison to Figure 4 and the biophysical parameter sensitivity analysis. The FWHM relative to both the instantaneous maximum and the global maximum were calculated to highlight the impulse response modulatory nature of TIRF and two-photon microscopy.

##### Lysosome binding

Datasets were collected via spinning disk confocal stimulation and imaging of cells co-expressing transiently transfected BcLOV4 and the LAMP1-miRFP670nano (Addgene plasmid #127435) lysosomal marker. 5 μM biliverdin was added to the imaging medium immediately before visualization to increase miRFP670nano fluorescence; non-specific partitioning of the cofactor into the plasma membrane was subtracted during analysis.

Models that accounted for the presence of lysosomes required the segmentation of lysosomal position from collected z-stacks (150 nm steps) of LAMP1-miRFP670nano fluorescence using a custom MATLAB script. In brief, the initial z-stacks were first de-convolved with the microscope PSF to reduce the impact of diffraction on downstream analysis. The xy-center of each lysosome was identified by sequential analysis of each 2D micrograph in the z-stack and by fitting each bright lysosomal puncta with a 2D Gaussian distribution (where the center of each distribution was the center of the corresponding lysosome). Each lysosome was visible in several z-stack frames despite the de-convolution step, and thus, its z-center was taken as the middle frame from the series of frames it appeared in. For practicality, the radius of all lysosomes was taken to be 340 nm (Woldemichael and Rosania, 2017). For theoretical simulations of the impact of lysosome volume density on the binding kinetics of BcLOV4, a mesh from an actual cell was randomly initialized with a given volume density of lysosomes and meshed as described previously.

##### Paradoxical fluorescence enhancement

In cases where BcLOV4 was imaged by a widefield microscope, an accurate 3D cell volume was absent and required volumetric extrapolation. To explore model accuracy for these conditions, videos of DMD-excited cells were collected for 1-10% duty cycles of stimulation (100 ms every 10 s, 250 ms every 10 s, 1000 ms every 10 s, or 1000 ms every 15 s). For each stimulated cell, a volumetric mesh was constructed by 3D extrapolated projection (as described in “Generation of volumetric cell mesh”), and 100 s of BcLOV4 spatiotemporal dynamics were simulated. To correct for photobleaching, biexponentials were fit for t > 150 s, i.e., when cellular concentration was at steady-state. To better visualize the recapitulation of paradoxical fluorescence enhancement within the stimulation ROI, the convolved model prediction was decomposed into its cytosolic and membrane components, since convolution is a linear operation.

To verify that the observed cytosolic fluorescence enhancement was truly an artifact of the diffractive properties of a widefield microscope, a similar experiment was conducted with DMD excitation and spinning disk confocal imaging. Specifically, data for DMD-excited cells was collected for 1-10% duty cycles of stimulation. In all cases, fluorescence enhancement within the excitation region was absent.

##### Cell morphology influence on association kinetics

Initially, meshes for spherical cells (r = 5-20 μm) with variable sized spherical nuclei (r = 1-10 μm) were generated. The resultant cell surface area-to-cytoplasmic volume ratios (SA/V) ranged from 0.15-1.2 μm^-1^. Simulations were then run on these geometries for 1 μM initial cytosolic BcLOV4 for duty cycles ranging from 0.67-10%, an excitation duration of 0.1 s with 405 nm light, and an excitation intensity of 12.24 W/cm^2^. The parameter τ_on_ was then extracted from resultant simulation data as the time constant of exponentials fit to cytosolic depletion curves and then compared to experimental data.

##### In silico transposition between experiments

Apparent τ_on_ for the cytosolic depletion method was calculated by fitting an exponential to the cytosolic decay curve. Apparent t_on_ for the colocalization method was calculated as described previously (Glantz et al., 2018); briefly, the correlation coefficient between a GFP-CAAX membrane marker and BcLOV4 was calculated over time for a 4 μm line profile perpendicular to the membrane. The appearance of a membrane virtual marker in the cell was simulated by setting the membrane to a uniform fluorescence intensity and setting the cytoplasm to a uniform intensity equal to 1/10^th^ that of the membrane. The latter 1/10^th^ value was calculated from a series of confocal images of GFP-CAAX and represents the cytosolic fluorescence that originates from marker protein that fails to traffic to the membrane. The simulated membrane marker image was convolved with the experimental PSF to account for diffractive effects before downstream analysis. τ_on_ could then be calculated by fitting an exponential binding curve to the relationship between correlation coefficient and time.

##### Virtual cell integration

The system from Equation 1 was input into Virtual Cell and a cell geometry composed of a nucleus, nuclear membrane, cytoplasm, plasma membrane, and extracellular space was also defined. A 3D cell mesh was constructed as described above (see “Generation of volumetric cell mesh*”*) and then converted to a stereolithography (,stl) file via custom MATLAB script for import into Virtual Cell. Similarly, the 3D excitation light distribution was computed as described in “Sensitivity to optical hardware diffraction.” To describe the volumetric spatiotemporal complexity of laser rastering, the resultant expressions were the scaled summation of multiple offset excitation PSFs, each offset by the spacing between raster points. A “light” input element was defined in Virtual Cell and assigned a value of either 0 when the light was off, or the aforementioned 3D light distribution expression when the light was on. The model could then be run to recover results similar to those of the FEM described herein.

We noted that differences between the Virtual Cell result and the result from our custom system largely stem from the coarser meshing required in Virtual Cell, which necessitates meshing of the extracellular space and is therefore less memory efficient. As a consequence of this coarser meshing, the modeled excitation volume is offset from its experimental location in space. To correct for this offset, the entire cell was shifted along the z axis so that the z-location (relative to the cell) of the simulated excitation volume was re-aligned to the experimental volume.

### Quantification and statistical analysis

Details of specific statistical analyses for each section, sample sizes, and statistical tests used are given in the STAR Methods and in the corresponding figure legends, but for completeness salient points are also summarized here. Notably, all analysis was done in MATLAB using custom written scripts, which are available with deposited data.

#### Confidence intervals and sample sizes for biophysical parameters

95% confidence intervals for calculated biophysical parameters (refer to Figure 2B) were generated by bootstrapping, using the MATLAB bootci() function with nboot = 1000. Sample size for: lit-state kinetic constants (n = 23 cells), dark-state kinetic constants (n = 10 cells), number of membrane binding sites (n = 83 cells), cytosolic diffusivity (n = 13 cells), and membrane diffusivity (n = 12 cells).

#### Confocal model accuracy comparison

The performance of the 3D non-linear model created herein were compared to those of a 3D linear model (described in “Simplified linear model”), a 2D non-linear model, and a 2D linear model. For the 3D non-linear and linear models, mesh generation proceeded as described above. For the 2D non-linear and linear models, the 2D segmented cell frame was directly meshed with the built-in MATLAB mesh generator. The models ran for 100 s for 17 different cells spanning 0.67–10% duty cycles (0.1 s stimulation per 1-15 s period) and were compared by the summed mean-squared error (MSE) between model prediction and experimental result over the entire time-course. Specifically, the MSE refers to error in spatial average cytosolic concentration of BcLOV4 between the model and experimental cell, taken over each time-point in the time-series. If we denote average model-computed cytosolic BcLOV4 concentration at time *t* as *f*_*m*_(*t*) and average measured experimental cytosolic BcLOV4 concentration at time *t* as *f*_*e*_(*t*), then the MSE represents the summation of (*f*_*m*_(*t*_*i*_) *– f*_*e*_(*t*_*i*_))^*2*^ over all timepoints *t*_*i*_ in the 100 s experiment (typically every 1 s) divided by the number of timepoints *i*.

For experimental data, the average over space was calculated by tracking average concentration over time within a 2 μm × 2 μm square region. This square region was selected based on requirements that it be sufficiently far from the large intracellular voids (i.e., nucleus) and from the membrane edge as to be unaffected by diffractive effects. Based on the PSF of the confocal microscope, the region was >1 μm away from any of these structures. The average fluorescence/concentration timeseries between different intracellular ROIs selected using these criteria tended to be similar (example shown in Figure S5C).

For 3D simulation data, the spatial summation was calculated by first generating 2D slices through the simulation volume at the same z-location as the cell focal plane. For 2D simulation data, this step could be skipped, as resultant simulation images were already 2D slices. The spatial averaging was then done by quantifying average concentration throughout the entire (now 2D) cell cytosol over time. Notably for simulation data, it was not necessary to track fluorescence within a matched ROI, since in the absence of simulated diffractive effects, there were negligible (<1%) subcellular differences in cytosolic BcLOV4 concentration in response to whole-cell stimulation. When analyzing imaging data where subcellular differences in cytosolic depletion may be notable (i.e., for patterned stimulation), comparisons between experimental and model results were done by tracking fluorescence between identical ROIs.

Note that all MSE comparisons were done between 2D images; 3D models were converted to 2D images before MSE analysis. Therefore the metric is equally meaningful for both 2D and 3D models. Statistical comparisons were conducted by paired Wilcoxon signed rank test. Significance was defined as p < 0.05.

## Data Availability

• Source data statement: Most of the raw data is available by Mendeley Data: https://doi.org/10.17632/62xbycxn4y.1. Due to size limitations, some microscopy data has been excluded, but will be shared by the lead contact upon request. • Code statement: The FEM toolbox is publicly available for MATLAB at Github: https://github.com/brianchowlab/BcLOV4-FEM and Zenodo: https://doi.org/10.5281/zenodo.6587636. A tutorial and examples are provided. The scripts used to generate the figures in this paper are available at Github: https://github.com/brianchowlab/reproducibility-BcLOV4-FEM and Zenodo: https://doi.org/10.5281/zenodo.6587665. • Any additional information required to reanalyze the data reported in this paper is available from the lead contact upon request.

## Supplemental information

Document S1. Figures S1–S7

Document S2. Article plus supplemental information
