# Supplementary material for: Computational framework for single-cell spatiotemporal dynamics of optogenetic membrane recruitment
Source: Cell Rep Methods. 2022 Jul 6;2(7):100245. doi: 10.1016/j.crmeth.2022.100245 (PMC9308134; doi:10.1016/j.crmeth.2022.100245)
Supplement: Document S1. Figures S1–S7 [file mmc1.pdf]

Cell Reports Methods, Volume *2*

Supplemental information

Computational framework for single-cell spatiotemporal dynamics of optogenetic membrane recruitment

Ivan A. Kuznetsov, Erin E. Berlew, Spencer T. Glantz, Pimkhuan Hannanta-Anan, and Brian Y. Chow

Supplemental figures and legends:

All described experiments were done with the BcLOV4-mCherry construct.

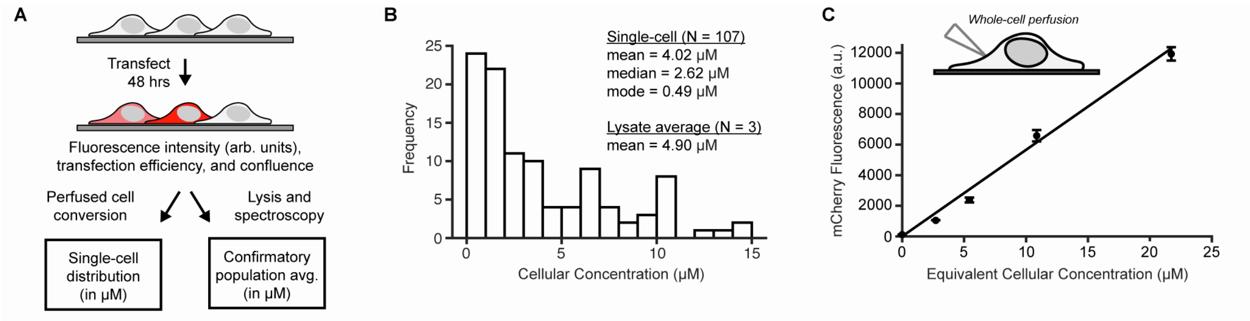

Figure S1. Quantification of absolute BcLOV4-mCherry cellular concentration in single cells; Related to Figure 2. Absolute concentrations (not relative fluorescence) are necessary to make bona-fide correlations between single-cell experimental data and their corresponding single-cell finite element models. **(A)** Schematic approach. Fluorescence imaging intensities of transfected cells were converted to absolute single-cell concentrations using a hardware-calibrated standard curve of fluorochrome-perfused cells (see panel C). The single-cell approach was cross-validated by the population average quantified by mCherry-fluorescence spectroscopy of lysate from trypsinized plates, assuming an average cell volume of 3 pL. **(B)** Single-cell distribution of intracellular BcLOV4-mCherry concentration. Cells most commonly showed relatively low, < 1 μM levels, of over-expressed protein in the rightward skewed distribution (N=107). The mean and median of the distribution are in reasonable agreement with the orthogonal population-level validation with lysate. **(C)** Calculated relationship between cellular mCherry fluorescence and intracellular concentration used to generate the single-cell distribution in panel B. Error bars represent ±3 standard deviations. Intracellular BcLOV4 concentrations were calculated from a standard curve of Lucifer Yellow dye-loaded cells (known intracellular concentrations by rapid perfusion via whole-cell patch micropipette) by normalizing for experimentally measured Lucifer Yellow::mCherry fluorescence ratios for the given optical system (N = 9, mean = 510.70 ± 11.14).

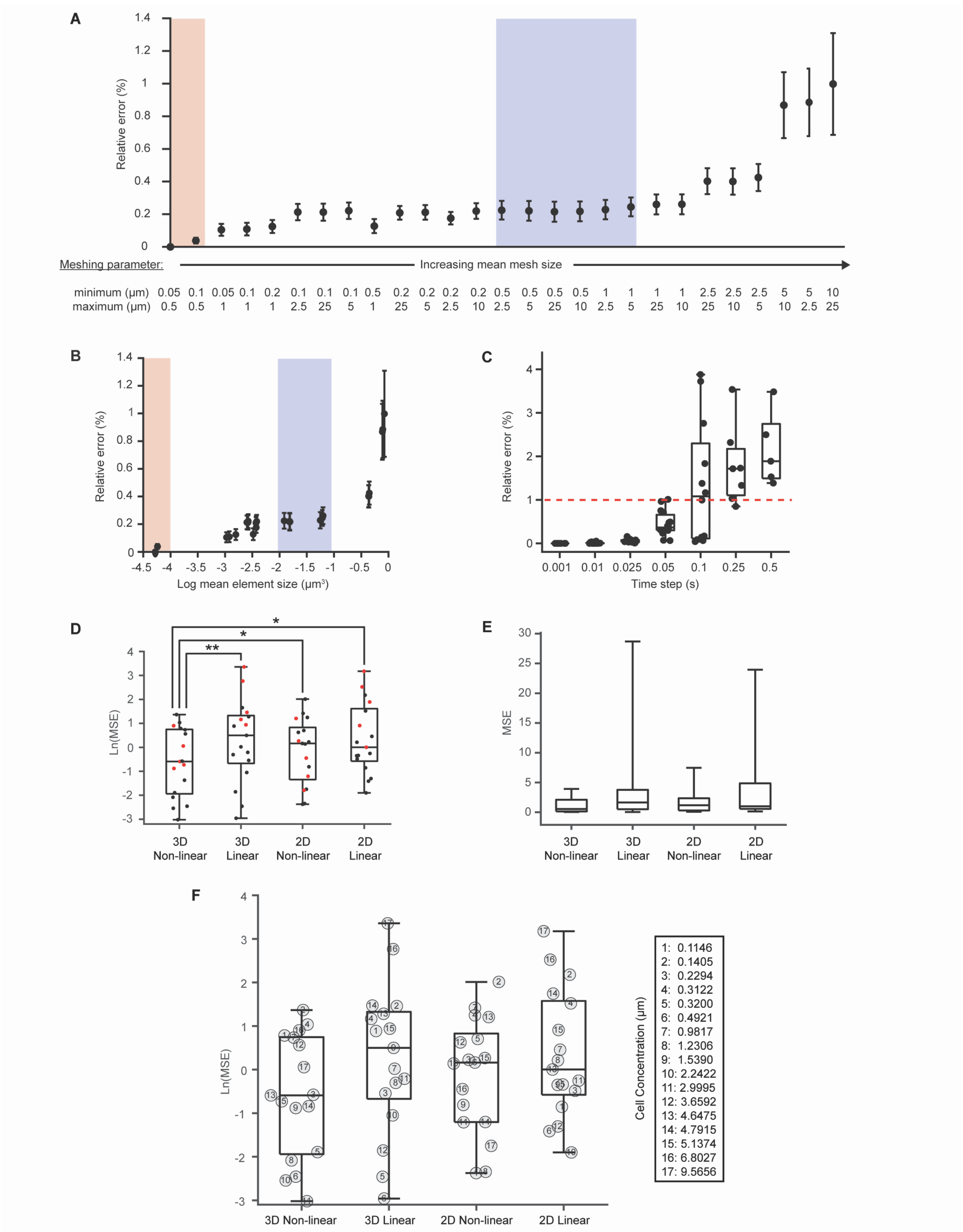

Figure S2. Effect of mesh size, model timestep, and initial concentration on error; Related to Figure 3. For mesh size and timestep, mean relative errors of < 1% were targeted. **(A)** Mean-squared error measured relative to finest mesh over a 100 s simulation, ordered by mean mesh size and indicated by

minimum and maximum length of each edge of tetrahedral elements, for wholefield stimulated cells (n = 12, error = standard error; 0.67%-10% duty cycle, 0.1 s pulse, 405 nm light, 12.24 W/cm^2). Blue area indicates mesh sizes used for typical simulations. Red area indicates finer mesh sizes to recapitulate the small excitation volumes of axial confined stimulation (**Figure 5D**) and small sizes of lysosomes (**Figure S6**). Less cells are included for mesh sizes > [2.5 μm minimum, 5 μm maximum; or mean element size ~1 μm^3) because convergence was not always achievable. **(B)** Results from panel (A) plotted with mean element size across all cells for each specific mesh min/max dimension. **(C)** Mean-squared error over a 100 s simulation for various timesteps, relative to simulation results for a 0.001 s timestep (n = 16 cells). Less cells are included for timesteps >0.1 s, because convergence was not always achievable, Red dotted line = acceptable error threshold used in this work. Initial BcLOV4 concentration was also investigated as a source of error. Refer to **Figure 3C** for mirrored data. Comparison of the mean-squared error (MSE) of the described 3D nonlinear model to the performance of a 3D linear model, a 2D nonlinear model, and a 2D linear model. Pooled data set contains cells stimulated at 0.67 - 10% duty cycles (or 0.1s pulse per 1-15s period), N=17. A paired Wilcoxon signed rank test was used for statistical comparisons of model performance. (*) $p < 0.05$, (**) $p < 0.01$. (D) Same plot as **Figure 3C**, with cells with the 5 highest intracellular BcLOV4 concentrations (>4 uM) shown in red. Linear models tend to perform worse for higher concentration cells. **(E)** Same plot shown with linear-y for alternate perspective of relative error spread. **(F)** Same plot showing error as function of model and of individual cell concentration, with each index corresponding to one cell of noted concentration.

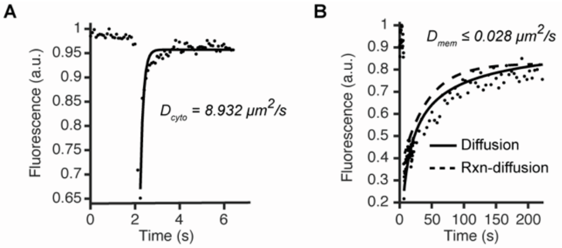

Figure S3: BcLOV4 diffusion constants determined by FRAP; Related to Figure 2. All experiments with BcLOV4-mCherry. **(A)** Cytosolic diffusion of dark-adapted BcLOV4-mCherry. Mono-exponential fit of recovery of fluorescence in region of interest (ROI), after bleaching with a λ = 561 nm laser. Model yields D_{cyto} = 8.932 μm^2/s. **(B)** Lateral diffusion along the membrane of photoactivated BcLOV4-mCherry after mCherry bleaching. Fit of recovery of fluorescence in ROI with a simple diffusion model (solid line; D_{mem} = 0.028 μm^2/s) and a reaction-diffusion model (dashed line; D_{mem} = 0 μm^2/s, $k_{off,lit}$ = 0.025 s^{-1}). The more conservative fit, of D_{mem} = 0.028 μm^2/s, is used for the model throughout this work.

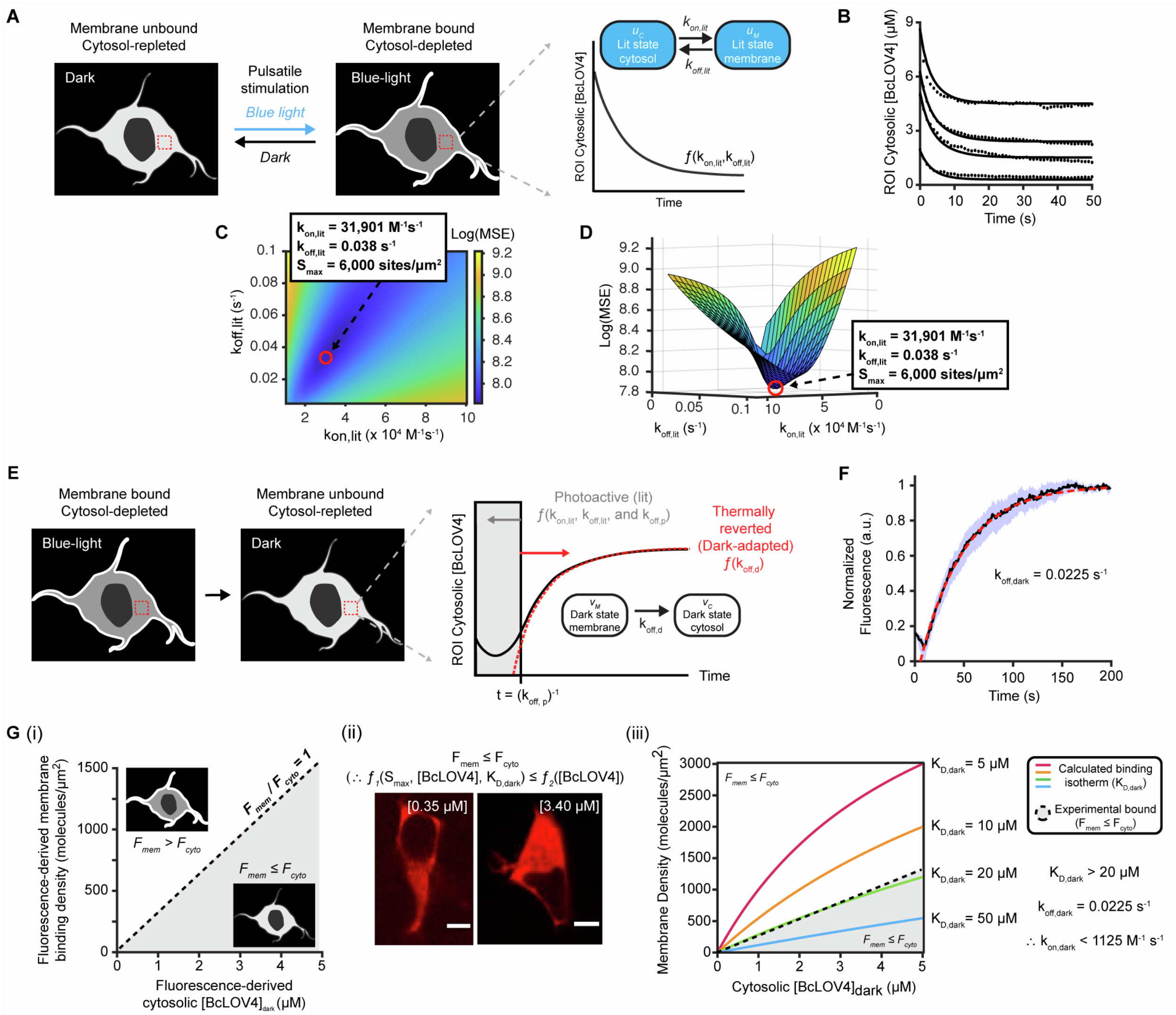

Figure S4: Determination of intracellular lit-state and dark-state membrane-binding kinetic constants from live cells; Related to Figure 2. All experiments with BcLOV4-mCherry. **(A)** Scheme for determining $k_{on,lit}$ and $k_{off,lit}$. HEK cells were stimulated for 100 ms (16.94 W/cm^2) every 1 second for 50 s total. Parameters were extracted from fits of the decrease in cytosolic fluorescence observed within a cytosolic ROI (red dashed box) that was >1 µm away from the membrane and nucleus. **(B)** Representative data (black dots) and the associated predicted fits (lines) for four cells. **(C)** Energy landscape heatmap showing mean-squared error of model versus experimental values for different values of $k_{on,lit}$ and $k_{off,lit}$ (N=23). The landscape is notably convex and converges to an apparent global minima at $k_{on,lit}$ = 31,901 M^{-1}s^{-1} and $k_{off,lit}$ = 0.038 s^{-1} (red circle). **(D)** 3D view of the energy landscape from panel B with minima indicated (red circle). **(E)** Schematized determination of $k_{off,dark}$ by post-illumination (5 s pulse, 16.94 W/cm^2) cytosolic repletion measurements in the dark for a given ROI (red box). After the photocycle or thermal reversion of the chromophore is complete ($t > 1/k_{off,p}$ = 18.5 s), the cytosolic fluorescence change is only dependent on $k_{off,dark}$ as the dark-state protein undocks from the membrane. **(F)** Experimental cytosolic fluorescence recovery (black line, 95% CI shading) for the measurement described in panel A, where $k_{off,dark}$ = 0.0225 s^{-1} (red). **(G)** Lower bound determination of $K_{D,dark}$ in live cells. Note that in the dark state $F_{mem} = f_1(S_{max},[\text{BcLOV4}]_{dark,KD,dark})$ and $F_{cyto} = f_2([\text{BcLOV4}]_{dark})$, where f_1, f_2 are functions which can be found experimentally (see **Methods**). Then, dark-state membrane fluorescence (F_{mem}) is lower than the cytoplasmic fluorescence (F_{cyto}) when $f_1(S_{max},[\text{BcLOV4}]_{dark,KD,dark}) \leq f_2([\text{BcLOV4}]_{dark})$. **(i)** Calibrated relationship between possible membrane-bound protein densities and cytosolic BcLOV4 concentration in dark-adapted state for the optical hardware (60x magnification spinning-disk confocal microscope). If

membrane localization is not apparent, the maximum membrane-bound protein density (black dotted line) can be estimated assuming $F_{mem} = F_{cyto}$. See **Methods** and **Figure S1** for F_{cyto}-derived quantification of $[BcLOV4]_{dark}$ in pipette-perfused cells and F_{mem}-derived quantification of surface density in liposomes devoid of diffractive confounds from the cytosol or interior bulk. **(ii)** Representative fluorescence micrographs of cells that meet the condition $F_{mem} \leq F_{cyto}$ or $f_1(S_{max},[BcLOV4]_{dark,KD,dark}) \leq f_2([BcLOV4]_{dark})$. Regardless of concentration, all cells observed in this study met this condition. **(iii)** Calculated binding isotherms (solid lines). $K_{D,dark}$ > 20 μM met the experimentally observed bounds (black dotted line, set from panel [C-i]), so it follows that $k_{on,dark} < 1125\ M^{-1}s^{-1}$.

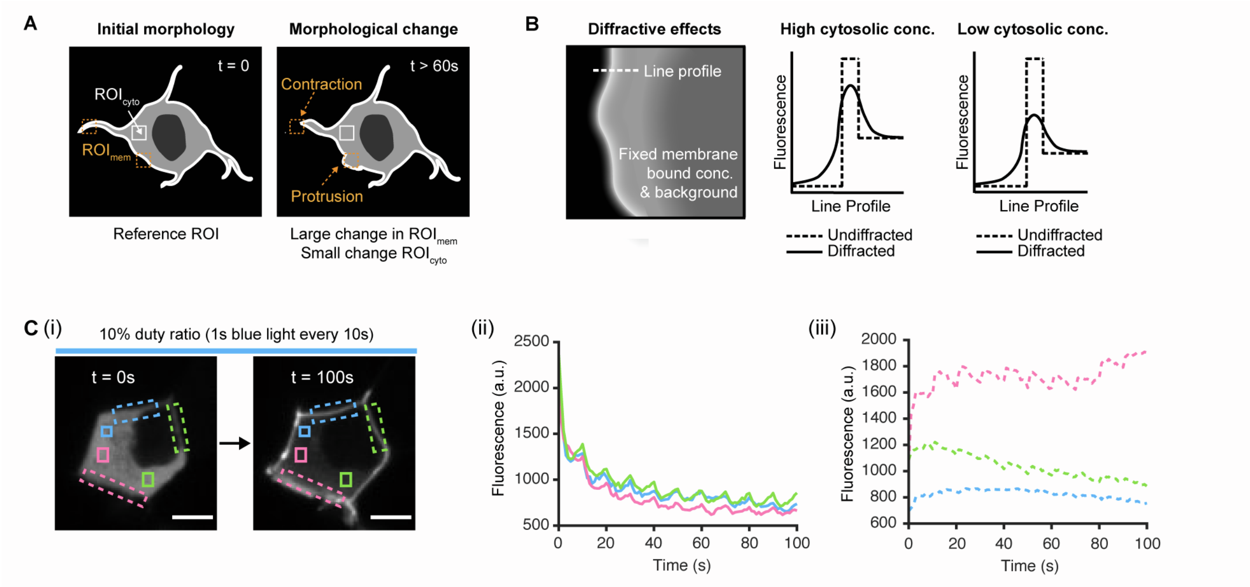

Figure S5: Variance of membrane protein density measurements derived from membrane fluorescence intensity; Related to Figure 3. All experiments with BcLOV4-mCherry. **(A)** Cell motility can change the spatial or morphological profile within a membrane region-of-interest (ROI) more so than the proximal cytosolic ROI. **(B)** Diffractive blurring of the plasma membrane and peri-membrane cytosol can cause cytosolic concentration-dependent inaccuracy in membrane density quantification from a line section. **(C)** Experimental demonstration of increased variability in membrane binding quantification by tracking the plasma membrane fluorescence vs. cytoplasmic fluorescence depletion. **(i)** Three quantification ROIs for a whole-field stimulated cell (dotted = membrane, solid = proximal cytosol). Scale bar = 5 µm. **(ii)** Cytosolic depletion measures from the ROIs are precise and accurate, including their tracking of binding/unbinding events in response to pulsatile stimulation. **(iii)** Plasma membrane ROI quantification is less precise and does not accurately capture the expected binding/unbinding cycle in all ROIs.

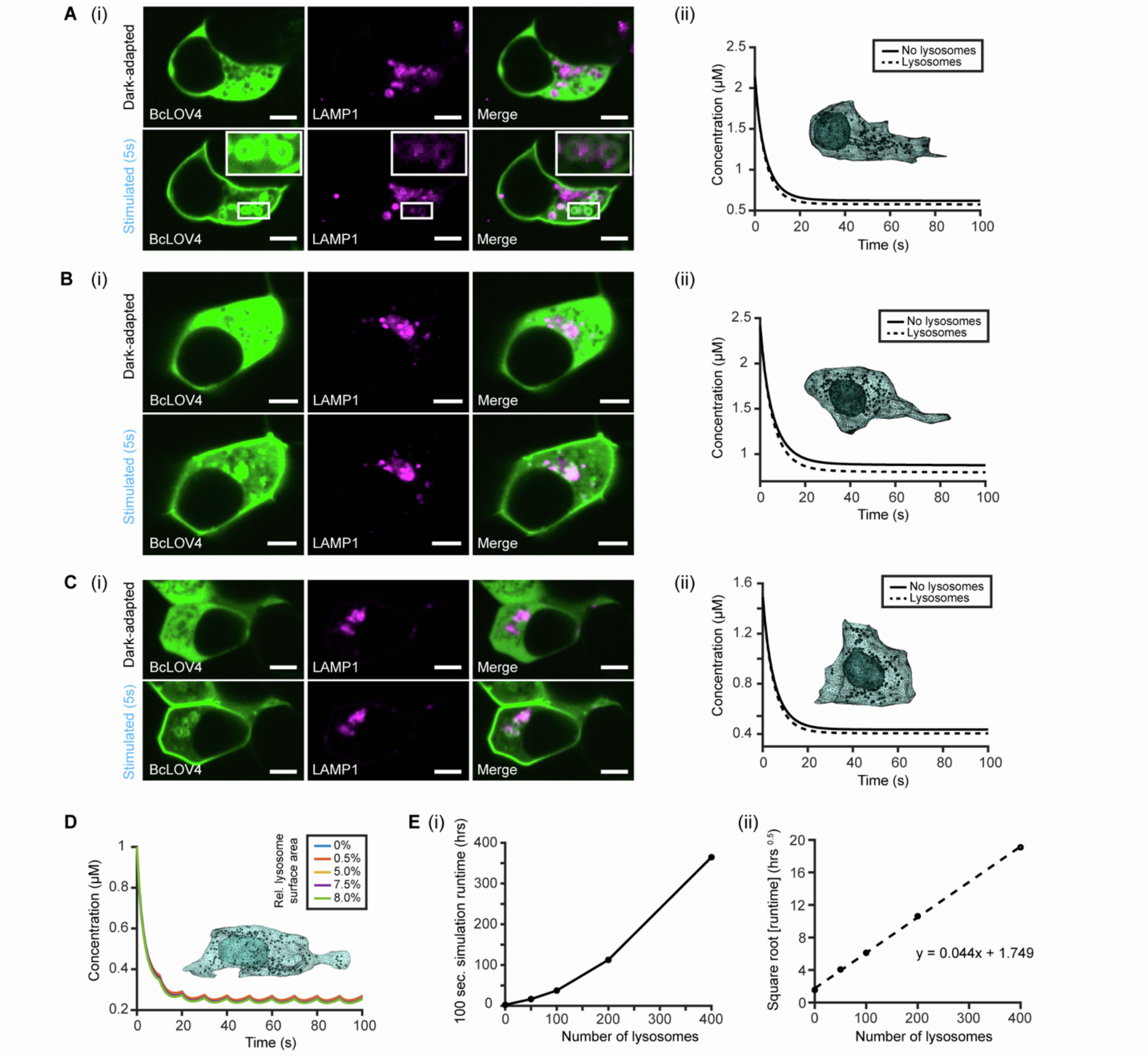

Figure S6. Lysosome contributions to single-cell FEM; Related to Figure 3. BcLOV4-mCherry expressing cells were photoactivated by pulsatile whole-field (unpatterned) stimulation with confocal imaging. **(A)** Dynamic recruitment in a representative cell with 0.70% lysosomes by volume. **(i)** Micrograph of BcLOV4 and co-localization to co-expressed lysosomal marker (LAMP1-miRFP670nano), before (t = 0 s) and after stimulation (t = 5 s). Scale bar = 5 µm. White box = inset area. **(ii)** Corresponding mesh reconstruction (inset, black dots = lysosomes) and model predictions for induced (0.1 s blue light pulses, 10% duty cycle, 12.24 W/cm^2) cytosolic depletion are closely aligned, regardless of accounting for lysosomes, suggesting that neglecting BcLOV4-lysosome interactions incurs minor error on the dynamics. Similar results for cells composed of **(B)** 0.72% and **(C)** 0.64% lysosomes by volume. **(D) (i)** Simulated recruitment of BcLOV4 to plasma membrane for different lysosomal surface areas (shown as percentage of plasma membrane surface area; 0.1 s blue light pulses, 2% duty cycle, 12.24 W/cm^2) for a sample cell (inset = mesh) with initial BcLOV4 concentration of 1 µM. Recruitment kinetics and steady-state value are relatively insensitive to increasing lysosomal fraction. **(E)** Computationally cost of accounting for lysosomes. **(i)** Runtime for 100 s-long single-cell simulated (with lysosome surface area ~0.05% that of the plasma membrane). **(ii)** Compute time scales O(n^2) with number of lysosomes.

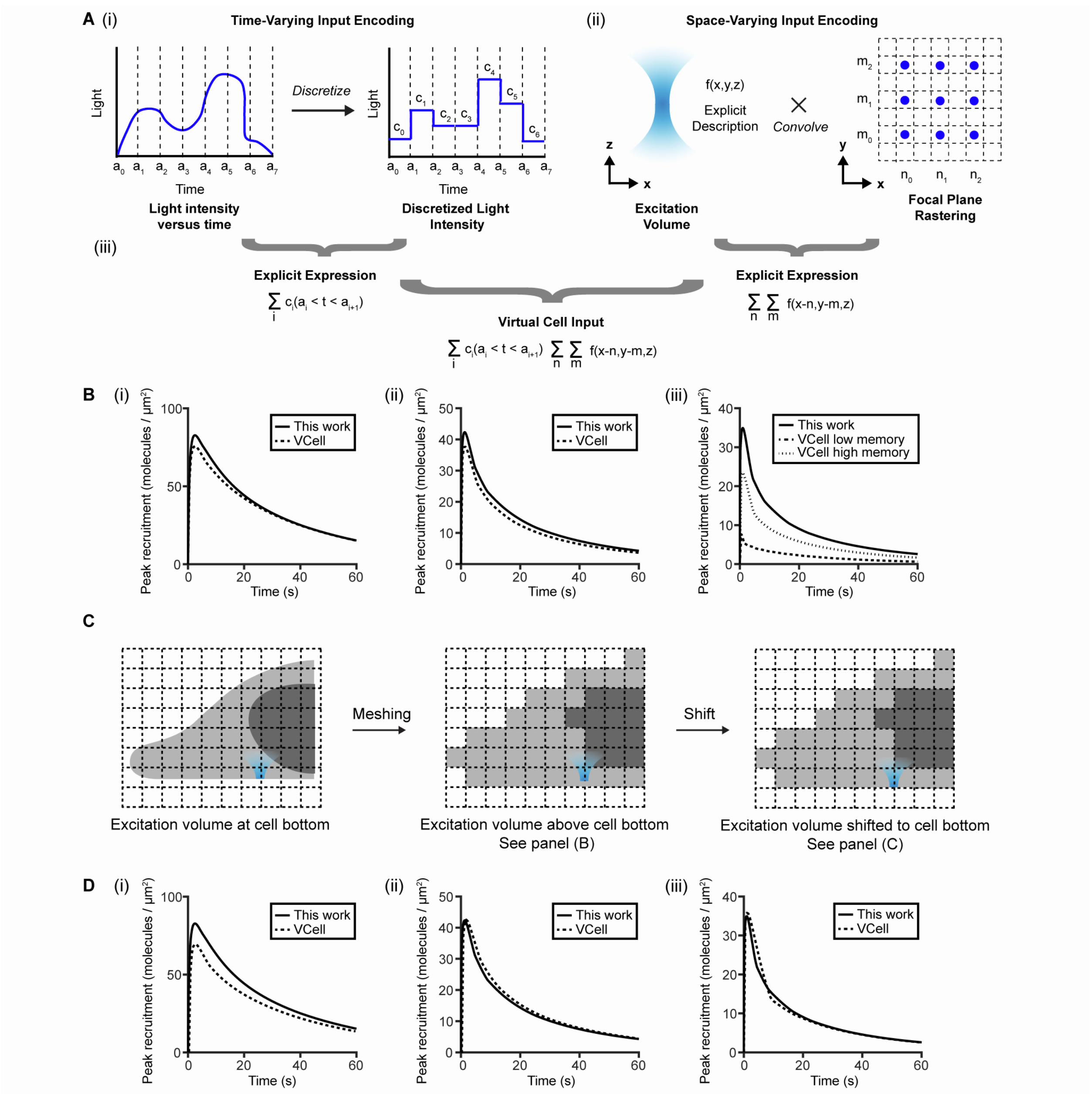

Figure S7. Modular workflow implemented in Virtual Cell; Related to Figure 5. **(A)** Generation of explicit representation of time- and space-varying inputs, as required by Virtual Cell (VCell). Decomposition of complex optical stimulation paradigms, like arbitrary laser focal-plane rastering patterns, into independent explicit representations of the temporal and spatial aspects that are combined post-hoc to generate the final Virtual Cell input expression. **(i)** Discretization of time-varying input expressions represented as the sum of Boolean logicals scaled by intensity within the given time-window. **(ii)** Explicit expression *f(x)* of the space-varying excitation volume approximated from microscope properties using physical optics or obtained by similar discretization as in (i), but now in space. *f(x)* is convolved with a map of raster points that the scanning-laser (or spinning-disk) illuminates at the focal plane to generate a space-varying expression. **(iii)** The expressions are multiplied to generate a time and space-varying expression. **(B)** Simulation of BcLOV4 membrane recruitment using described model (solid line, from **Figure 6D-ii)** and Virtual Cell (dotted line) for the geometry and excitation in **Figure 6D-i** by **(i)** 1-photon (1P), **(ii)** 2-photon (2P), and **(iii)** TIRF stimulation (2 µm x 2 µm region, duration = 100 ms, 10 W/cm^2 irradiance at the focal plane, [BcLOV4] = 1 µM for all methods). Note the model and Virtual Cell agreement for 1P/2P excitation but large discrepancy for TIRF. Discrepancy between models for TIRF excitation originates from meshing.

Virtual Cell uses a finite volume-based method of uniform orthogonal mesh, resulting in domain surface conversion to a “staircase” representation that can functionally alter the cell geometry and shifts the bottom of the cell, thus creating an offset between cell bottom and focal plane that is dependent on mesh fineness. Virtual Cell requires a rectangular prismatic extracellular mesh, resulting in coarser meshes due to memory limitations, which becomes apparent for the small excitation volumes of TIRF. The practically capped maximum memory requirement of 16 Gb RAM resulted in typical mesh element volumes of ~0.015 μm^3 for the Virtual Cell simulations and 0.002-0.005 μm^3 for simulations the custom FEM. **(C)** Partial correction of offset by shifting stimulated excitation volume along with the mesh and **(D)** simulation comparison using the shift-correction. **(i)** 1P, **(ii)** 2P, and **(iii)** TIRF. The correction method does not preserve distance relations between the excitation volume and intracellular structures (e.g. altered overlap between nucleus and excitation volume).
